# Sustainable Application of Waste Sludges from the Wastewater Treatment Plant Generated during the Production of Heating Devices in the Construction Industry

**DOI:** 10.3390/ma17051089

**Published:** 2024-02-27

**Authors:** Marija Stojmenović, Jelena Gulicovski, Neda Nišić, Nenad Ristić, Shanke Liu, Jorge Loredo, Milan Kragović

**Affiliations:** 1 “Vinča” Institute of Nuclear Sciences, National Institute of the Republic of Serbia, University of Belgrade, 22-24 Mike Petrovića Alasa, 11351 Belgrade, Serbia; rocenj@vin.bg.ac.rs (J.G.); neda.nisic@vin.bg.ac.rs (N.N.); m.kragovic@vin.bg.ac.rs (M.K.); 2 Faculty of Civil Engineering and Architecture, University of Niš, Aleksandra Medvedeva 14, 18106 Niš, Serbia; nenad.ristic@gaf.ni.ac.rs; 3 Key Laboratory of Mineral Resources, Institute of Geology and Geophysics, Chinese Academy of Sciences, Beijing 100029, China; liushanke@mail.iggcas.ac.cn; 4 Department of Mining and Exploration, University of Oviedo, C. San Francisco, 3, 33003 Oviedo, Asturias, Spain; jloredo@uniovi.es

**Keywords:** industrial waste sludge, mortar, concrete, self-compacting concrete, sustainability, recycle

## Abstract

This research presented, for the first time, the results of the successful application of the waste press sludges, WSLP (plant for lacquer and paint) and WSEP (powdery enamel plant), from a wastewater treatment plant generated during heating device production in the construction industry. The results of WSEP characterization and its influence on cement paste, mortar, and concrete properties showed that this material could be used as a cement replacement (with a maximum replacement amount of 20%) in producing mortar and concrete. Although waste WSLP sludge does not possess pozzolanic properties and does not meet the criteria prescribed by the standards for application in mortar and concrete due to its chemical inertness and fineness, as well as its extended setting time, it can be used as a replacement for stone filler or other powdered mineral admixture in the production of self-compacting concrete (SCC) in amounts up to 100%, with a maximum quantity of up to 100 kg/m^3^. The obtained results indicate that with the appropriate conversion, waste sludges, despite representing hazardous waste, can be used as safe products in the construction industry; i.e., the waste material can become a useful and valuable raw material by applying (respecting) all of the principles of the green economy.

## 1. Introduction

Rapid population growth, urbanization, and technological advances, which all lead to progressive industrialization, are the main initiators of the issues related to the generation of immense amounts of harmful waste worldwide. The disposal of such waste materials, which are potentially toxic and hazardous, is a major global concern due to the negative environmental impacts [1,2]. Due to the increasing generation of waste over the years during production in various industrial processes, governments have recently faced rather strict protocols on environmental protection and legal norms related to final waste management, as well as to the reduction in emissions and total air, water, and soil pollution that may occur. The essence of such increasingly strict regulatory requirements lies in the tendency of modern society and scientific circles to contribute to the transition towards a circular economy [3,4,5] and the establishment of a sustainable development concept by applying so-called zero-waste strategies.

Since the 1970s, the concept of the circular economy (CE) has been gaining attention among the academic community. It emerged as the opposite economic model to the traditional linear economy, favoring the elimination of end-of-life concepts and forming sustainable production systems based on closing the material and energy loops. This shift was achieved by reducing the use of primary resources and promoting the recycling, reprocessing, and valorizing of waste materials [6,7]. In this context, the fundamental goal today is the transformation of recyclable wastes into valuable alternative materials that can promote sustainability in different fields of industry. With the introduction of these alternative utilization routes for waste materials, not only the mitigation of the issues related to the scarcity of appropriate disposal sites but also certain economic and ecological benefits and significant reductions in energy consumption can be provided [8,9]. The significant advantages of turning waste into a value-added resource, as one of the central pillars of the CE model, involve reducing the costs related to the management and disposal of waste as well as the costs that arise from environmental regulations and taxes, and providing positive ecological outcomes due to pollution alleviation. Moreover, substituting more expensive virgin materials with widely available waste could reduce the final product price and achieve primary resource conservation [5]. All of the above-mentioned factors explain why the interest of the contemporary world in developing new alternative practices for waste application has been significantly rising recently.

Also, water consumption, as an essential utility and resource for sustainable development and a necessary resource in all industrial sectors, is directly linked with the quantity of generated wastewater that must be treated and decontaminated [10]. Therefore, industrial wastewater treatment plants are considered to be significant generators of large amounts of solid waste, predominantly in the form of wastewater sludge, also referred to as “residuals” [11]. These waste products must also be strictly regulated and adequately managed for environmental protection. Moreover, the CE business model has become especially popular concerning operations in the wastewater sector [5,7]. Therefore, it is necessary for water utilities to fully adopt this concept to manage their resources and develop sustainable strategies.

On the other hand, innovative and eco-friendly routes for manufacturing sustainable and efficient building materials have been attracting considerable attention recently, and this has significantly intensified since the construction sector represents one of the largest and fastest developing sectors in the world economy [12]. Therefore, the construction industry has appeared as one of the most appropriate destinations for using waste products such as different types of wastewater sludge. In the research carried out so far, it has been determined that sludge generally has a low density, which makes it a suitable and valuable component in the production processes of various types of light construction materials. Most of the previous research refers to the application of waste sludge in producing bricks, lightweight aggregates, pavements, ceramic products, etc. [13]. For instance, many studies [8,14,15,16,17,18] have reported favorable results from applying wastewater sludge as an alternative raw material, which can partially substitute commercial clay as a traditional basic component in the production of bricks without damaging the bricks’ mechanical integrity. Moreover, some papers, such as those of Limami et al. [17] and Santos et al. [18], have shown that fired brick samples produced with a wastewater treatment plant sludge content of up to 20% exhibited improved thermal performance with higher specific heat capacity. Additionally, in the study conducted by Trang et al. [18], it was successfully demonstrated that wastewater sludge can even be reused as a component for adobe (unburnt) bricks made using hydraulic press technology. Manufacturing adobe bricks by incorporating waste sludge and additives like polypropylene fiber can enhance their quality and structure, offering a more affordable alternative to traditional fired bricks [18]. Besides, many scientific articles propose the incorporation of wastewater sludge in the industrial production of ceramic tiles for various purposes, such as glazed, roof, and floor tiles. Considering the results of mechanical properties, water absorption, apparent density, and the other features obtained in some of the studies [19], sludge can be successfully added even at a percentage above 30% of the total final tile mix with no detrimental effects on the performance. More interestingly, Rodrigues and Holanda [20] concluded that integrating waste sludge as a replacement for kaolin by up to 10% can allow the production of ceramic floor tiles at lesser firing temperatures, which might lower manufacturing costs and have significant financial benefits in addition to the environmental ones.

However, the extensive literature overview evidenced that many recent publications have focused on studying the perspective of sludge from the water treatment process for different ways of its application in the cement and concrete industry. Regarding this, it is important to note that almost all developed countries worldwide depend on concrete as one of the basic engineering materials in most building structures. Also, the concrete industry is one of the largest consumers of virgin raw materials of nature and, therefore, has a bad reputation in terms of ecology [19]. Moreover, concrete prices have risen drastically in the past decade due to high demand and production costs. This was an additional initiative for researchers to find a way to utilize waste sludges as alternative raw materials for concrete production and potentially solve this crisis.

The outcomes of the many research studies of different authors, summarized in Table 1 [9,21,22,23,24,25,26], reported that wastewater sludge and similar by-products can be considered as beneficial environmentally risk-free raw materials that may be integrated into the cementitious matrix and be a partial substitute for sand in cement mortar or cement concrete mix. To that end, a practical and feasible utilization solution can be accomplished with the possibility of being quickly commercialized.

However, as seen in Table 1, most studies refer to the waste sludge generated in municipal wastewater (i.e., sewage) treatment plants, which may be ascribed to a less complex and potentially problematic composition than the industrial wastewater sludge type. Given the heterogeneity and complexity of physical and chemical properties as well as the possible presence of certain traces of heavy metals, toxic materials, and organic pollutants, wastewater sludge produced by processes involved in various industry sectors, such as the chemical industry, may be considered as a highly challenging waste stream [7]. Therefore, current attempts to recycle such waste products are somewhat deficient. Thus, the literature available in this research field so far is limited.

To expand the research scope in this area, the present paper analyzes the possible utilization of sludges from the treatment of industrial wastewater as an efficient and eco-friendly construction material additive. It suggests returning it to the production chain as a valuable resource. It is worth mentioning that the company’s main activity is producing heating devices on solid, liquid, and gaseous fuels, electric stoves, and a combination of solid-fuel, electricity–gas, and electricity. Therefore, it should be emphasized that the wastewater in this plant originates from different, rather complex chemical processes, which may indicate the potentially problematic composition of the obtained waste sludge. Accordingly, mechanical and physicochemical properties were examined in detail to confirm whether the prescribed criteria can be met in producing concrete and mortar for cement that is partly replaced by waste.

Thus, the main goal of this paper was to investigate the possibility of transforming industrial wastewater sludges generated during the production process of heating devices into value-added products in the form of fabrication components of different construction materials. More precisely, this study investigates the potential reuse of waste sludges in their raw state as a partial replacement for cement in preparing concrete and mortars. Incorporating wastewater sludge into the production of some construction materials, such as concrete, would provide the safe insertion and dispersion of small but not negligible amounts of presented heavy metals in the cement matrix. That would prevent possible harmful effects of their toxicities on human health and ecosystems [27,28]. Additionally, the proposed solution for the final waste management follows current legal requirements, the significant principles of sustainability, and the zero waste strategy, which alleviates adverse environmental impacts and provides some financial benefits.

## 2. Materials and Methods

### 2.1. Materials

**Figure 1:**
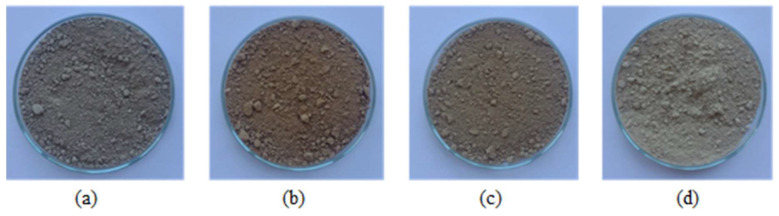
WSLP generated in: (**a**) October; (**b**) November; (**c**) December (2019); and (**d**) January (2020).

**Figure 2:**
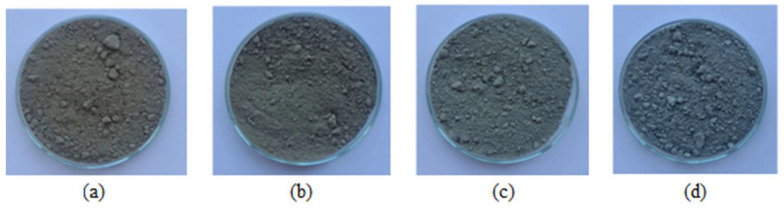
WSEP generated in: (**a**) October; (**b**) November; (**c**) December (2019); and (**d**) January (2020).

In recent years, different companies have aimed to reduce the amount of freshwater used during the production process and the amount of generated wastewater, sludge, and solid waste. For example, in the last four years, the companies producing heating devices in the Republic of Serbia generated about 160 tons of the mentioned waste, representing a quantitatively large proportion of potentially unused material. Therefore, the aim is to use the waste categorized as non-hazardous (“Rulebook on the categories, testing and classification of waste (Sl. glasnik 56/2010, 93/2019, 39/2021)”) as a raw material in the construction industry. During October (2019), November (2019), December (2019), and January (2020) the following waste materials were generated and sampled (Figure 1 and Figure 2):

WSLP—waste sludge from the treatment of lacquer and paint wastewater—press sludge;

WSEP—waste sludge from the plant for wastewater treatment from powdery enamel plant—press sludge.

### 2.2. Characterization Methods

#### 2.2.1. X-ray Powder Diffraction

The X-ray powder diffraction (XRPD) of polycrystalline materials was applied to determine the phase composition of samples. Scanning was performed on a diffractometer for powder samples, which is a part of the SIEMENS D500 automated system (Cu—Kα_1,2_ radiation, λ_1,2_ = 1.54184 Å), in the 2θ range of 20–75°, with a step size of 0.02°/s. The diffractograms were graphically presented and analyzed using Diffrac*^plus^* software v1.1. The Eva program, a component of this software, facilitated several essential tasks, including the removal of the Cu—Kα2 X-ray contribution from reflections, determination of reflection intensities and 2θ angles, calculation of the d-spacing values for crystal planes defined by Miller indices (hkl), and measurement of the width of diffraction profiles for reflections. The search/match feature allowed for comparing diffractograms obtained from samples with a comprehensive database. PDF (powder diffraction file) cards published by the Joint Committee on Powder Diffraction Standards (JCPDS), now known as the International Center for Diffraction Data (ICDD), were employed for crystal phase identification. 

#### 2.2.2. Determination of the Heavy Metal Content

The heavy metal content of waste sludges WSLP and WSEP was determined by inductively coupled plasma optical emission spectrometry (ICP-OES). An ICP spectrometer Spectroflame (Spectro Analytical Instruments, Kleve, Germany) was used for the analysis. However, concentrations of arsenic were obtained in other ways by microwave digestion using the EPA 3051A procedure.

#### 2.2.3. Determination of the Specific Surface Area

The BET method was applied to obtain the investigated samples’ specific surface. For this analysis, adsorption and desorption isotherms of adsorbed gas, nitrogen N_2_, at a temperature of liquid nitrogen (−196 °C) under static conditions were determined as a function of relative pressure. It was assumed that gas adsorption occurs in each sample’s monomolecular layer. Measurements were performed using the Micromeritics ASAP 2020 instrument. Before analysis, the samples were degassed at 120 °C and vacuumed for 24 h. According to the obtained results, the adsorption isotherms were plotted using the BET equation. The amounts of adsorbed gas in the monomolecular layers of nm were determined from the slopes and sections of the linear parts of the adsorption isotherms. Then, the values of the specific surface areas of the samples Sp (m^2^/g) were calculated. From certain isotherms of all samples, the following transparency parameters were calculated: V_total_—total pore volume; V_meso_—mesopore volume (according to the αs method); V_micro_—micropore volume (according to the BJH method from the desorption branch of the isotherm); D_sr_—the mean pore diameter; as well as D_max_—the diameter of pores that occupy the most of the volume.

#### 2.2.4. Fourier-Transform Infrared Spectroscopy with Attenuated Total Reflection

Attenuated total reflection (ATR) represents a method used in addition to Fourier-transform infrared spectroscopy (FTIR) and allows the examination of solid or liquid samples directly, without any particular preparation. The FTIR spectrum of samples was recorded using the Thermo Scientific Nicolet iS10, FTIR Spectrometer (Thermo Fisher, Waltham, MA, USA) with a recording range of 4000–400 cm^−1^ at ambient temperature to provide information on the type of functional groups on the surface of waste materials.

#### 2.2.5. Field Emission Scanning Electron Microscopy

Surface topography testing was conducted on the samples to explore the morphology and microstructure of the resulting waste materials. The microstructure was examined using a MIRA3 TESCAN scanning electron microscope (Tescan, Kohoutovice, Czech Republic), with secondary electron imaging (SEI) employed for capturing FE-SEM images. An accelerating voltage of 10.0 kV and ×10 k magnification were applied for the analysis. The samples were prepared by coating with a nanometer-thick golden layer (a few nm) using the Fine Coat JFC—1100E ION SPUTTER JEOL.

### 2.3. Investigation of the Pozzolanic Activity, Cement Paste Parameters, Mortar, Concrete, and Self-Compacting Concrete Produced with Addition of Waste Sludges

#### 2.3.1. Investigation of the Pozzolanic Activity and Cement Paste Parameters with Addition of Waste Sludges

To determine the potential use of waste sludges WSLP and WSEP as type II admixtures in making concrete following the standard EN 206, the samples generated in October, November, December 2019, and January 2020 were taken and tested. To determine the possibility of applying the mentioned sludges WSLP and WSEP in the production of mortar and concrete, an examination of the pozzolanic activity and parameters of cement paste with the addition of waste sludges was carried out by the following methods: pozzolanic materials class (SRPS B.C1.018) [29], activity index (EN 450-1) [30], water requirement (EN 450-1 Annex B), standard consistence (EN 196-3) [31], initial setting time, a final setting time (EN 196-3), and soundness (EN 196-3). The detailed procedures of each applied method according to the corresponding EN standard are presented in Supplementary Materials S1.

#### 2.3.2. Investigation of the Mortar Parameters Based on Portland Cement, Natural Sand, and the Admixture of Waste Sludge WSEP

To test the parameters of mortar mixtures consisting of Portland cement, natural sand, and a certain amount of waste sludge WSEP, the samples generated in October, November, December 2019, and January 2020 were taken. A total of 5 different mixtures were made with waste sludge WSEP: an etalon mixture as reference mix-design (marked with E; Table S1) and four mixtures in which the partial replacement of cement by sample WSEP was carried out in the amounts of 7.5%, 15%, 22.5%, and 30% by weight (marked as WSEP-7.5, WSEP-15, WSEP-22.5, WSEP-30; Table S1). For mortar preparation, fine river sand (fraction 0/2 mm) was used with a specific gravity of 2610 kg/m^3^ and water absorption of 1.52%, Portland cement CEM I 52.5 R with a specific gravity of 3.15 g/cm^3^ and a specific surface area of 4020 cm^2^/g, waste sludge WSEP, water, and chemical additive type superplasticizer. By the implementation of the different methods, the following properties were determined: consistency (EN 1015-3) [32], bulk density of fresh (EN 1015-3) and hardened mortar (EN 1015-10) [33], mechanical strength—flexural (EN 196-1; EN 1015-11) [34,35] and compressive strength (EN 196-1; EN 1015-11), water absorption at atmospheric pressure (EN 13755) [36], water absorption (determination of water absorption coefficient due to capillary action of hardened mortar; (EN 1015-18) [37], drying shrinkage (SRPS B.C8.029:1979; ASTM C 596) [38,39], adhesion of a concrete substrate (determination of adhesive strength of hardened rendering and plastering mortars on substrates; EN 1015-12) [40], and leaching test (EN 12457-2) [41]. The detailed procedures of each applied method according to the corresponding standard are presented in Supplementary Materials S1.

#### 2.3.3. Investigation of the Concrete Parameters Based on Portland Cement, Natural Sand, Coarse Crushed Aggregate, and Added Waste Sludge WSEP

To test the parameters of concrete mixtures consisting of Portland cement, coarse crushed aggregate, natural sand, and waste sludge WSEP in precisely defined percentages, the sample generated during November 2019 was taken and tested. Four different mixtures in total were prepared: an etalon mixture (labeled E; Table S2) and three mixtures in which the partial replacement of cement by waste sludge WSEP was carried out by weight of 10%, 20%, and 30% (marked WSEP-10, WSEP-20, and WSEP-30; Table S2). For making concrete, a crushed gabbro with fractions 4/8 mm and 8/16 mm was used as the coarse aggregate with a specific gravity of 2890 kg/m^3^ and water absorption of 0.58%; the fine aggregates were river sand with a fraction of 0/4 mm, the specific gravity of 2620 kg/m^3^, and water absorption of 1.42%, ordinary Portland cement CEM I 52.5 R with a specific gravity of 3.15 g/cm^3^ and specific surface area 4020 cm^2^/g, waste sludge WSEP, water, and chemical additive-type superplasticizer. Determination of the different properties was performed using the following methods: consistency—slump test (EN 12350-2) [42], air content in fresh concrete (EN 12350-7) [43], density of fresh (EN 12350-6) [44] and hardened concrete (saturated with water; EN 12390-7) [45], mechanical strengths—flexural (EN 12390-5) [46], compressive strength (EN 12390-3) [47], and tensile splitting strength (EN 12390-6) [48], elasticity secant modulus (EN 12390-13) [49], depth of water penetration under elevated pressure (EN 12390-8) [50], freezing and thawing resistance with deicing salts (CEN-TS_12390-9) [51], rebound number (EN 12504-2) [52], and ultrasonic pulse velocity (EN 12504-4) [53]. The detailed procedures of each applied method according to the corresponding standard are presented in Supplementary Materials S1.

#### 2.3.4. Investigation of SSC Parameters Produced with the Addition of Waste Sludge WSLP

Since the research showed that the waste sludge WSLP does not show pozzolanic activity and therefore does not satisfy the criteria related to the activity index according to standard EN 450-1 (Section 4.1), its further examination in order to ascertain its potential application was carried out using it as a substitute for stone filler in the self-compacting concrete (SCC) testing. To test the parameters of self-compacting concrete (SCC) with the waste sludge WSLP used as a replacement for limestone in a defined percentage (100%), the sample generated in November 2019 was investigated. Two SCC mixtures were made: a reference mixture (marked E-SCC; Table S3) and one where the limestone filler was replaced with waste sludge WSLP (marked WSLP-SCC; Table S3). Tested SCC mixtures were made with natural river aggregate (sand) with grain size 0–4 mm, large, crushed aggregate (fractions 4/8 mm and 8/16 mm), Portland cement CEM I 52 R, waste sludge WSLP, lime-stone filler, water, and chemical superplasticizer-type admixture. The parameters of SCC concrete were examined under the following methods: slump flow test (EN 12350-8), T500 test (EN 12350-8), L-box passing ratio determination (H2/H1) (EN 12350-10) [54], testing of segregation using sieves (EN 12350-11) [55], air content determination in fresh concrete (EN 12350-7), density of fresh (EN 12350-6) and hardened concrete (water saturated; EN 12390-7) evaluation, determination of mechanical strengths—flexural (EN 12390-5), compressive (EN 12390-3), and tensile splitting strength (EN 12390-6), elasticity secant modulus (EN 12390-13), depth of water penetration under elevated pressure (EN 12390-8), freezing and thawing resistance with de-icing salts (CEN-TS_12390-9), determination of rebound number (EN 12504-2), and testing of ultrasonic pulse velocity (EN 12504-4). The detailed procedures of each applied method according to the corresponding standard are presented in Supplementary Materials S1.

## 3. Results and Discussion

### 3.1. XRPD

**Figure 3:**
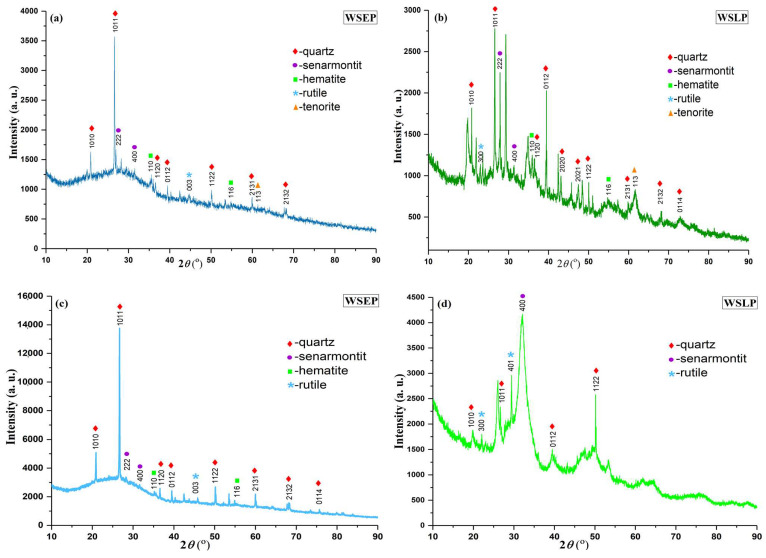
XRPD diagrams of the waste sludges: (**a**) WSEP and (**b**) WSLP generated in October 2019; (**c**) WSEP and (**d**) WSLP generated in January 2020.

X-ray diffraction analysis was used to determine the phase composition of all waste sludges as an indication of the formation of solid solutions. Figure 3 shows the XRPD diagrams for the representative waste sludges WSEP and WSLP generated in October 2019 and January 2020, respectively. Based on the obtained qualitative mineralogical analyses, it was determined that quartz (SiO_2_) mineral is the most present in crystal phases of both groups of samples. In contrast, as side minerals, oxides of Sb, Fe, Ti, and Cu in the form of senarmontite (Sb_2_O_3_), hematite (Fe_2_O_3_), rutile (TiO_2_), and tenorite (CuO) are presented. In addition to identified peaks, XRPD diagrams show unidentified peaks, as well as noticeable differences in the width of the diffraction peaks between sludges, which can be due to the increase in crystal size due to the development of different chemical reactions and the influence of temperature during the production process. It should be kept in mind that the process of production of heating devices is a very complex chemical process that takes place under the influence of different temperatures and which can lead to the formation of various compounds (chemical or ionic) of non-stoichiometric composition, whose peaks using the XRPD method are challenging to define due to the limitations of the applied database.

### 3.2. Chemical Composition

According to “Rulebook on the categories, testing and classification of waste (Sl. glasnik 56/2010, 93/2019, 39/2021)”, as well as EN 12457/EN 16192: 2011, which are applied in the Republic of Serbia, in each investigated sample of waste sludges WSEP and WSLP the concentrations of certain heavy metal ions exceed the maximum permissible values for classification of waste as non-hazardous (Table 2); therefore, these waste sludges must be considered as hazardous waste. For using waste materials in the construction industry, the restrictions prescribed by the “Rulebook on restrictions and prohibitions on the production, placing on the market and use of chemicals (Sl. glasnik 90/2013, 25/2015, 2/2016, 44/2017, 36/2018, 9/2020, 57/2022)”, as well as by the “Rulebook on the categories, testing and classification of waste (Sl. glasnik 56/2010, 93/2019, 39/2021)” are in use. However, no limits are defined for the application based on the concentration of heavy metals in waste materials.

Further, analyzing the content of oxides in the composition of waste sludges WSEP and WSLP, it can be noted that SiO_2_, Sb_2_O_3_, CaO, MgO, TiO_2_, MnO_2_, Fe_2_O_3_, Co_3_O_4_, CuO, and Al_2_O_3_ are dominant in sludge WSEP. In contrast, sludge WSLP mainly contains SiO_2_, CaO, MgO, Fe_2_O_3_, and Al_2_O_3_, but the content of SiO_2_ is five times lower than in sludge WSEP (Table 3). It is a well-known fact that to be used in the production of mortar and concrete, the powdery materials must be pozzolanic active, for which the presence of high amounts of SiO_2_, Fe_2_O_3_, and MgO is necessary. Although the mentioned oxides were recorded using the XRPD method, the SiO_2_ content is only satisfactory in the WSEP sludge. On the other hand, the test results showed that although the Fe_2_O_3_ and MgO oxide content is higher in the WSLP sludge, the SiO_2_ content is five times lower. That gives guidelines on the potential possibility of limiting the application of this sludge in the construction industry due to low or completely absent pozzolanic activity, which agrees with the analysis of the results of pozzolanic activity presented in Section 3.1.

### 3.3. The Specific Surface Area

**Figure 4:**
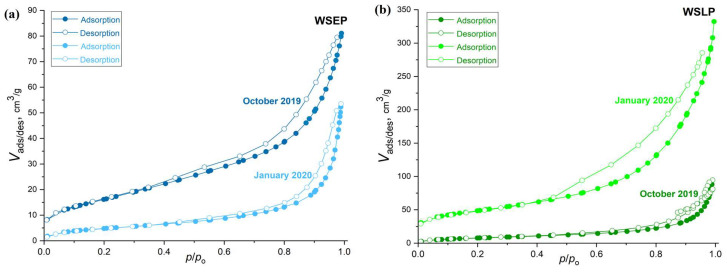
N_2_-physorption isotherms of the waste sludges: (**a**) WSEP generated in October 2019 and January 2020; (**b**) WSLP generated in October 2019 and January 2020.

According to the IUPAC classification [57], obtained nitrogen isotherms of investigated waste sludges WSEP and WSLP belong to type IV (Figure 4). A hysteresis loop was noted (Figure 4), characteristic of mesoporous materials, while a large volume suggests the presence of micropores in all samples adsorbed at low relative pressures. The values of the total specific surface areas (*Sp*) of waste sludges WSEP and WSLP generated in the raw state were measured and calculated using the BET equation based on the noted adsorption nitrogen isotherms (Figure 4).

For the WSEP and WSLP generated in October and November 2019, Sp values are in the range of 29–60 m^2^/g (Table 4), while values for the waste sludges WSEP and WSLP generated in December 2019 and January 2020 are in the range of 18–168 m^2^/g (Table 4). For the samples, the following parameters were additionally calculated: Vtotal—total pore volume, V_meso_—mesopore volume, pores between 2 and 300 nm, V_micro_—micropore volume, D_sr_—mean pore diameter, and D_max_—pore diameter occupying most of the volume (Table 4; Supplementary Materials S2).

Lower values of specific surface areas of sludge WSEP and WSLP samples agree with their higher agglomeration degrees. Mesopores generally form between particles, while micropores are on the particles themselves. When comparing the obtained results (Table 4), it can be concluded that different agents in the production process ultimately yield different effects of decreasing or increasing the surface area, depending on the specific production procedure. The agents in the complex production process hamper the formation of the ordered structure in the self-assembly step, which can result in the overall reduction in the surface area. The values of the porosity parameters ultimately depend on the specific conditions of the production procedure. According to the criterion prescribed by Standard EN 13263-1 [58], the specific surface area of the samples of waste sludges WSEP and WSLP should be above 15 m^2^/g, so these waste materials could be used as an admixture of type II for the production of concrete following EN 206. Therefore, from the results obtained, it can be concluded that generated waste sludges WSEP and WSLP meet the criteria for use as type II additives for concrete production following EN 206. However, the relatively high surface areas and significant contribution of mesopores of waste sludge WSEP and WSLP can affect the parameters of the investigated mortar and concrete, which we address in the following sections.

### 3.4. FTIR

**Figure 5:**
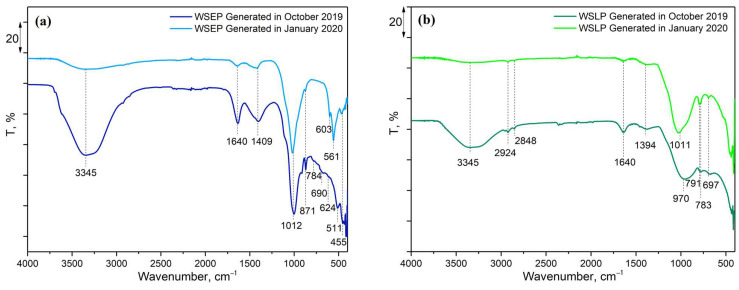
FTIR spectra of the waste sludges: (**a**) WSEP and (**b**) WSLP, generated in October 2019 and January 2020.

To investigate the structural properties of the waste sludges WSEP and WSLP, Fourier-transform infrared spectroscopy (FTIR) followed by attenuated total reflection (ATR) was used. The spectra for four months for both sludges were determined, and results for WSEP and WSLP generated in November 2019 and January 2020, as the most representative, are shown in Figure 5. According to the obtained results, it may be observed that there were no significant deviations in structural properties between waste sludge samples generated in different months.

For sludge WSEP (Figure 5a), the following spectral bands were recorded: 455, 511, 561, 603, 624, 690, 784, 871, 1012, 1409, 1640, and 3345 cm^−1^. The spectral bands at 455, 511, and 1012 cm^−1^ originated due to silicon, present in the SiO_2_ form [59] characteristic of the Si–O or Si–O–Si group vibrations. The band at 603 cm^−1^ originated due to the presence of Sb, in the form of senarmontite (Sb_2_O_3_), and originated due to Sb–O–Sb vibration [60], while the band at 561 cm^−1^ is a consequence of the presence of Fe in the form of hematite (Fe_2_O_3_) and the stretching vibration of the Fe–O [61]. The spectral band characteristic for TiO_2_ is located at 690 cm^−1^ and is a consequence of the Ti–O stretching vibration [62]. The spectral bands at 784 and 624 cm^−1^ are characteristic of CuO and correspond to the Cu–O stretching vibrations in the monoclinic structure of the tenorite [63]. The absorption bands at 3345 and 1640 cm^−1^ originated from the –OH stretching vibration and HOH bending mode of adsorbed water molecules [63], while the band at 1409 cm^−1^ is due to the presence of the –OH group of the adsorbed water molecules and its stretching vibrations. The absorption band in the range 850–900 cm^−1^ indicates that Mg is present in the sludge and is characteristic of cubic MgO [64].

For sludge WSLP (Figure 5b), similar spectral bands were obtained as for sludge WSEP, with a slight shift in the spectral band’s positions and intensities. This sludge’s spectral bands were noted: 697, 783, 791, 1011 (970), 1394, 1640, and 3345 cm^−1^. The bands at 3345, 1640. and 1394 cm^−1^ can be assigned to the -OH stretching and banding vibrations of the adsorbed water molecules [63]. The band at 1011 (970) cm^−1^, as well as the doublet at 791 and 783 cm^−1^, are proof of the presence of SiO_2_ [59,65], while the band at 697 cm^−1^ is due to the presence of TiO_2_ [62]. In contrast to WSEP, in the spectrum of the WSLP sludge, the presence of doublet spectral bands at 2924 and 2848 cm^−1^, which are typical for vibrations of the C–H group in alkanes indicates that an organic alkane component is also present in this sludge. Considering that the intensity of these lines is small, it can be concluded that the share of organic components is limited and insignificant [66].

Considering that the spectral bands characteristic for mentioned chemical species (SiO_2_ and TiO_2_) in the WSLP sludge are significantly wider compared to the WSEP sludge, the bands characteristic of CuO, MgO, Sb_2_O_3_, and Fe_2_O_3_, which were also detected in this sample by XRPD analysis, could not be identified due to overlapping.

Additionally, in both sludges, there are no visible bands that could be used to define and determine the presence of other oxides of elements determined by classical chemical analysis given in Table 2, which can also be explained by the fact that the content of oxides of other elements is significantly lower compared to those detected by XRPD analysis, and their characteristic spectral bands due to low intensity could not be detected by FTIR analysis. Also, it can be noted from Table 2 that the content of some elements in examined samples is relatively high, such as of Al, Mn, and Ca, but, at the same time, these elements were not detected by FTIR analysis. An additional explanation is that these elements are most likely in cations or metallic forms (with zero valence), known to be undetectable by FTIR analysis [67].

### 3.5. FE-SEM

**Figure 6:**
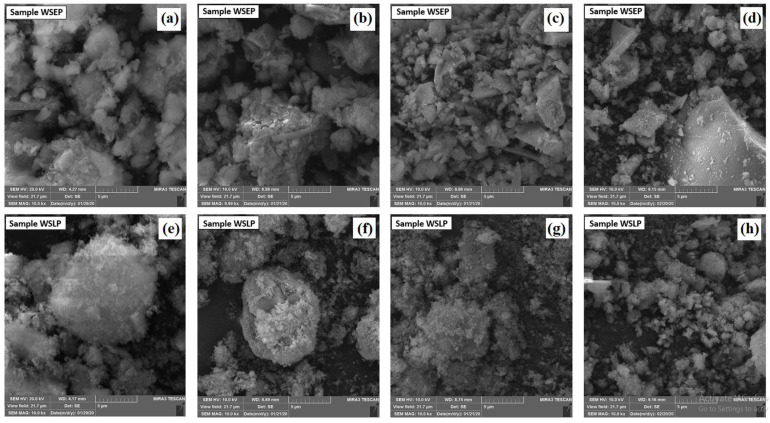
FE-SEM micrographs of the waste sludge WSEP generated in: (**a**) October 2019; (**b**) November 2019; (**c**) December 2019; and (**d**) January 2020, and WSLP generated in: (**e**) October 2019; (**f**) November 2019; (**g**) December 2019; and (**h**) January 2020.

Micrographs obtained using field emission scanning electron microscopy (FE-SEM) are shown in Figure 6. Observation of the microstructure of the generated waste sludges WSLP and WSEP shows the appearance of different polygonal crystals in all samples and a certain degree of agglomeration. More uniform distribution and smaller size of particles, as well as higher porosity (Table 4), are characteristic of waste sludge WSLP and WSEP samples from wastewater treatment plants generated in December 2019 and January 2020 (Figure 6). On the other hand, agglomeration processes are very often present in the process industry, whether it is wanted agglomeration through a production process or unwanted agglomeration like, for example, caking, which can also be seen in Figure 6. In general, it can be concluded that applying different specific conditions of the production procedure can influence the degree of agglomeration.

## 4. The Pozzolanic Activity, Cement Paste Parameters, Mortar, Concrete, and SSC Concrete Produced with Addition of Waste Sludges

### 4.1. The Pozzolanic Activity and the Impact of Cement Replacement by Waste Sludges WSEP and WSLP on the Cement Paste Properties

To investigate the potential application of waste sludges WSEP and WSLP generated during October, November, December 2019, and January 2020 as a type II admixture for concrete production according to standard EN 206, the class of pozzolanic materials and activity index were initially determined. The results are presented in Table 5 and Table 6.

It was determined that waste sludge WSEP samples possess pozzolanic properties (the class of pozzolanic materials 5; Table 5) and, therefore, meet the criteria for testing the activity index according to EN 450-1 concerning the possibility of using the material as a type II admixture for concrete production following EN 206. The soundness condition stipulated by the same standard is also met, while the proscribed standard regarding water requirement and initial setting time is not satisfied. Replacement of cement with 25% of waste sludge WSEP caused an increase in the standard consistency of cement paste. Given that the value of a low class of pozzolanic activity of waste sludge WSEP, the substitution of cement with waste sludge WSEP during the production of concrete and mortar for further research was in the range of 7.5–30%. Detailed results of testing the effects of partial substitution of cement with waste sludge WSEP on the concrete and mortar parameters are presented in Section 4.2 and Section 4.3.

On the other hand, it was determined that the waste sludge WSLP possesses no pozzolanic activity and does not meet the activity index criteria following EN 450-1 (Table 6). Concerning the water requirement and the initial setting time, the conditions prescribed by the same standard are not met, while the condition for soundness is fulfilled (Table 5). The replacement of cement with 25% of waste sludge WSLP greatly influenced the increase in the standard consistency of the cement paste. However, due to its chemical inertness and fineness, sludge WSLP can be used as a replacement for stone filler (or other powdered mineral admixture such as fly ash, red sludge, ground granulated blast furnace slurry, and silicate dust) in the production of self-compacting concrete (SCC). So, waste sludge WSLP was further used as a substitute for stone filler in the self-compacting concrete (SCC) testing, and the detailed results are presented in Section 4.4.

### 4.2. Mortar Parameters Based on Portland Cement, Natural Sand, and Added Waste Sludge WSEP

**Figure 7:**
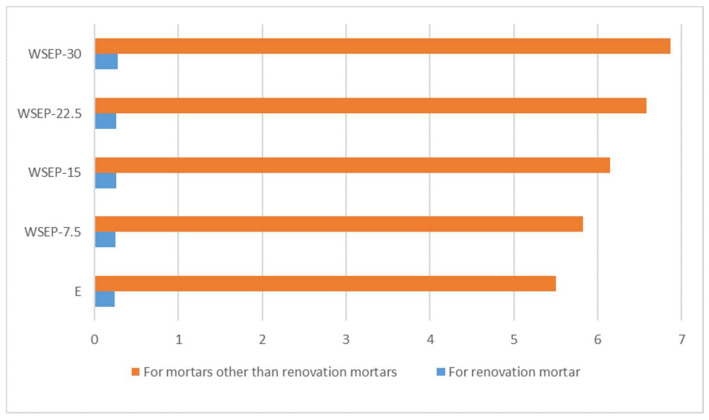
Water absorption coefficient due to capillary action—C [kg/m^2^].

**Figure 8:**
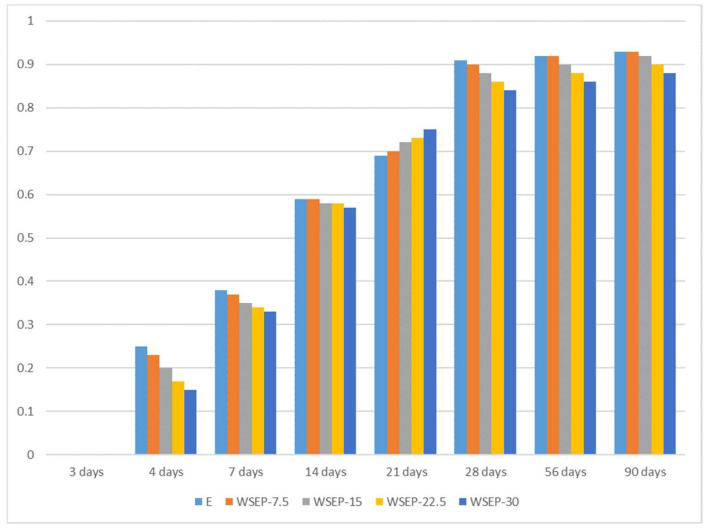
Shrinkage due to drying over a period of 90 days—***ɛ_sm,sr_*** [mm/m].

As the most representative, the results of the analysis of mortar mixture with the addition of waste sludge WSEP generated in November 2019 are shown in Table 7 and Figure 7 and Figure 8. The results of the mortar mixture with the addition of waste sludge WSEP generated in other months are presented in Supplementary Materials S3, Tables S6–S8, and S12–S17.

**Figure 9:**
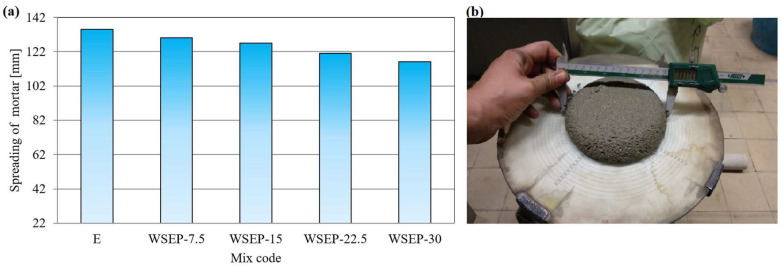
(**a**) Spreading of mortar on the table; (**b**) visual representation of mortar consistency testing by flow table.

According to the presented results (Table 7; Figure 7 and Figure 8), the mortar consistency with the addition of waste sludge WSEP meets the criteria prescribed by the standard and is similar to the value obtained for the etalon sample. During consistency testing, the mortar slump on a shaking table decreased with the increase in the share of cement replaced with waste sludge WSEP. That can be explained by the increased water requirement of waste sludge WSEP, which reduces the amount of free water in the mortar and its plasticity. A graphical representation and a visual display of the results of mortar consistency testing are given in Figure 9a,b. In addition, the results of the replacement of cement with increasing the content of waste sludge WSEP indicate a slight decrease in the fresh and solid mortar bulk density. Possible reasons are the lower specific mass of sludge WSEP than of cement, as well as the decreased plasticity and, consequently, increased porosity of the mortar.

**Figure 10:**
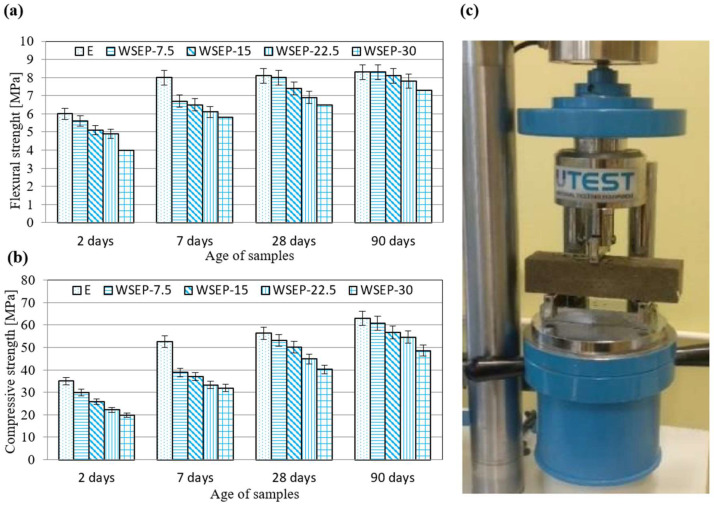
(**a**) Flexural strength of mortar samples; (**b**) compressive strength of mortar samples; (**c**) visual representation of testing mortar flexural strength.

Mechanical strengths, especially compressive ones, are significant parameters for cement composites. The flexural and compressive strength were determined for the mortar specimens aged 2, 7, 28, and 90 days. A graphical and visual representation of the results are presented in Figure 10a–c. As observed, with an increase in cement replacement by waste sludge WSEP, the strength of mortar specimens decreases at early ages (2 and 7 days; Table 7). That can be attributed to the lack of cementitious action on higher percentage replacement. This indicates that at the very beginning, CaO is present in the waste sludge; WSEP does not take part in the cementitious action [68,69,70]. Heavy metals such as zinc and lead also form a protective coating around cement grains, preventing them from participating in the hydration reaction [71]. For the mentioned process, the normal hydration process is hindered because of a characteristic reaction between heavy metals and significant cement hydration compounds, mainly in the form of dicalcium silicate and tricalcium silicate. For example, a membrane of calcium hydroxyl zincate (CaZn_2_(OH)6⋅2H_2_O) can coat the surface of the cement compounds and prevent the cement hydration reaction and the movement of required water. In such a way, the presence of heavy metal content in the WSEP sludge can particularly retard the normal hydration process and ultimately lower the compressive strength values of mortars [71,72,73]. However, the mentioned heavy metals in the WSEP sludge are not present in large quantities, so it can be considered that their influence is noticeable only at early ages (2 and 7 days).

**Figure 11:**
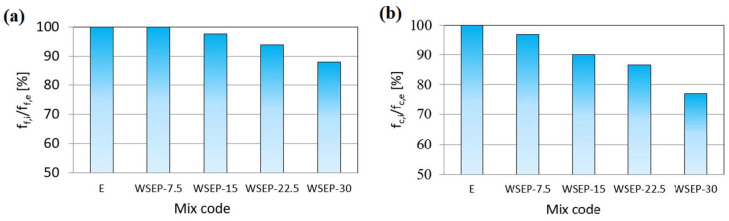
Percentage ratio of: (**a**) flexural and (**b**) compressive strength of mortar with waste sludge WSEP and reference mortar at 90 days.

On the other hand, with increasing age (28 and 90 days), the strength of mortar samples approaches the values of the etalon mortar. This can be explained by the pozzolanic reaction of waste sludge WSEP, which is triggered at a later hardening stage. For this reason, the possible share of cement replaced with waste sludge WSEP is estimated following the sample’s strength at 90 days. Figure 11 represents the ratio of flexural (11a) and compressive strength (11b) of the mortar prepared with partial substitution of cement with waste sludge WSEP and the reference mortar sample (etalon) at the age of 90 days. As the diagrams show, the decrease in flexural strength that occurs with an increase in the percentage of cement replacement by waste sludge WSEP is less pronounced than the decrease in compressive strength. Considering the acceptable level of strength decrease of 15%, it can be claimed that in mortar production, up to 20% of cement can be successfully replaced with waste sludge WSEP.

Further, cement replacement by waste sludge WSEP increased the water absorption value, regardless of the test method. Water absorption at atmospheric pressure is at least on the etalon mortar, while with the increase in cement replaced by waste sludge WSEP, the value of absorption increased by max. 23%. A similar trend is observed when determining the capillary absorption of water. The lowest absorption coefficient was observed in the etalon mortar and the highest in the mortar labeled “WSEP-30”.

Based on the measurements of shrinkage happening as a result of drying cement mortars, it can be observed that mortar mixtures had lower shrinkage rates in the initial curing period (first 7 days) in comparison with etalon mortar. At day 14, all mortar mixtures had similar values of shrinkage. In the aging period from 7 to 21 days, higher shrinkage was observed in the mortars with the waste sludge WSEP, while at the later phase of curing (from 29 to 90 days), the reference mortar had the highest shrinkage after drying. A lower shrinkage of mortar with the waste sludge WSEP results from its porosity and water absorption capacity, which prevents the loss of free water from the mortar structure. The cement replacement with sludge WSEP also affected adhesion and caused a slight reduction in the mortars for the concrete base. With the increase in the percentage of cement replacement, the adhesive strength decreases in the range from 5% to 30%. The lower limit of adhesive strength value for repair mortars amounts to 1.5 MPa, and all tested mortar mixtures except “WSEP-30” satisfy this requirement.

The concentration of elements in water after leaching and the maximum permitted values provided for in the “Rulebook on permitted quantities of hazardous and harmful substances in soil and irrigation water and their testing methods (Sl. glasnik 23/1994-553)” are presented in Table 7. The leaching test results showed that the peak levels of all health-based contaminants were within the respective safety limits specified in the “Rulebook on permitted quantities of hazardous and harmful substances in soil and irrigation water and their testing methods (Sl. glasnik 23/1994-553)”. The compliance with Rulebook Sl. glasnik 23/1994-553, according to safety limits, indicates that the application of sludge WSEP should not significantly affect human health or endanger the environment.

The results clearly show that it is possible to achieve successful mortar production using the waste sludge WSEP based on its composition of different inorganic and organic compounds. By analyzing WSEP sludge using different methods, clear guidelines for its application directly in mortar production as a raw form, without additional treatment and cost, were obtained. The development of technically feasible processes for mortar production and the application of different sludges were mainly limited due to higher production costs (if compared to market price) and weakness of economic profitability, which was overcome when applying waste sludge WSEP. Furthermore, using sludge WSEP as a raw material in mortar manufacturing significantly reduces the burden from natural resources like clay and limestone (source of CaO, SiO_2_, and F_2_O_3_) [74]. According to the present testing results of the mortar, WSEP sludge can successfully replace ordinary Portland cement up to 20% by weight. This research gives promising output worldwide regarding the use of waste sludge from the production process of heating devices in the construction industry for the first time.

### 4.3. Parameters of Concrete Based on Portland Cement, Natural Sand, Large Crushed Aggregate, and Added Waste Sludge WSEP

Since the parameters of waste sludge WSEP for all batches generated during October, November, and December 2019, as well as January 2020, were continuously determined, only the results for mixtures with the addition of waste samples generated in November 2019, as the most representative, are shown. Using the methods mentioned in Section 3, concrete mixtures were prepared by combining Portland cement, large crushed aggregate, natural sand, and waste sludge WSEP added in precisely defined percentages of 10%, 20%, and 30% (samples labeled as WSEP-10, WSEP-20, and WSEP-30). Table 8 shows the composition of concrete mixes and the testing results on fresh and hardened concrete.

**Figure 12:**
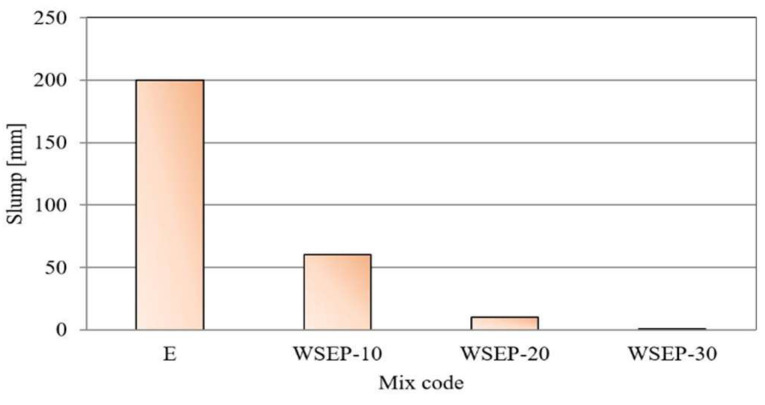
Concrete slump measuring results.

As the results suggested, increased amounts of waste sludge WSEP in concrete caused a signified decrease in its consistency. That may be justified by waste sludge WSEP’s porosity and its water retention ability. Therefore, reducing the free water in the concrete mixture leads to a lower consistency. There was no slump in the mixture with 30% of waste material. A graphical representation of concrete consistency results obtained by the slump test method is given in Figure 12.

**Figure 13:**
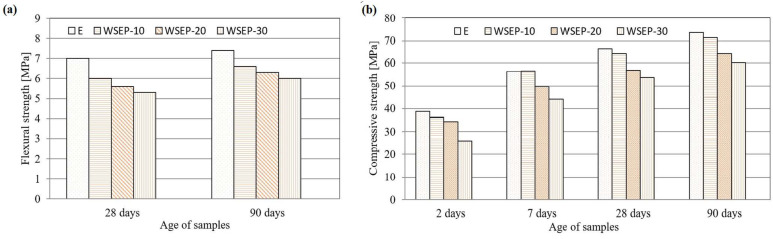
(**a**) Flexural strength of concrete; (**b**) compressive strength of concrete samples.

The substitution of cement with waste sludge WSEP caused the improper change in the values of the bulk densities of concrete in the fresh and solid state. However, these changes range within ±1%, which is considered acceptable. The air content that can be drawn into fresh concrete decreases with the increase in the percentage of cement replacement with waste sludge WSEP. The etalon concrete mixture displays the highest percentage of entrained air (2.6%), while for the mixture “WSEP-30,” the lowest value (1.8%) is measured. This can be explained by the better concrete placement with the addition of waste sludge WSEP. The flexural strength was determined for samples aged 28 and 90 days. The results are graphically presented in Figure 13a. It may be observed that the flexural strength increases with the increase in the share of cement replacement, and these values are in comparison with reference concrete in the range from 11 to 19% for the sample aged 90 days. Compressive strength accounts for the most important property of concrete, and it is determined for specimens at ages of 2, 7, 28, and 90 days (Figure 13b). At first, it can be stated that with the increase in the share of cement replacement by waste sludge WSEP, the concrete specimens’ strengths decrease regardless of age. In the initial 7 days, the highest increase in compressive strength is measured for the reference mortar, while with the increasing age of samples, the compressive strength of concrete prepared with waste sludge WSEP approaches the reference concrete mixture because of the delayed reaction of pozzolanic activity.

**Figure 14:**
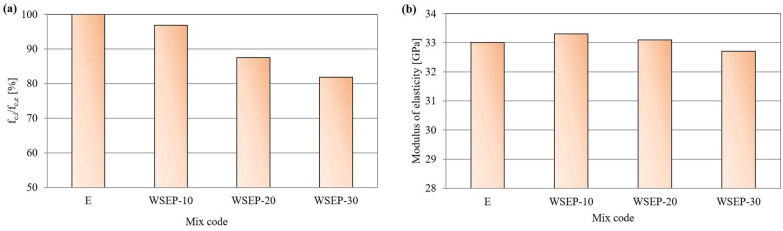
(**a**) Percentage ratio of compressive strength of concrete with waste sludge WSEP and reference concrete at 90 days; (**b**) secant modulus of elasticity of concrete.

Figure 14a shows the ratio of percentages of compressive strength of concrete prepared with partial cement replacement by waste sludge WSEP and the reference concrete sample at 90 days. From the following diagram, a slight decrease in compressive strength with an increase in the percentage of cement replacement by waste sludge WSEP can be seen. Since 15% is an acceptable rate of compressive strength reduction, it can be concluded that WSEP can replace cement up to 20% in concrete production. The decrease in tensile splitting strength occurs with the increase in the share of cement replaced by waste sludge WSEP (Table 8). The highest tensile splitting strength value (3.9 MPa) is recorded for reference concrete, while the concrete mixture with a maximum (30%) of cement replacement displayed 18% lower tensile splitting strength. The elasticity secant modulus of concrete was determined for a sample at the age of 28 days. As Figure 14b shows, the modulus of elasticity of concrete increases with the increase in the percentage of cement replaced with waste sludge WSEP up to 10%. The maximum value reached is 33.3 GPa. With a further increase in cement replacement, the modulus of elasticity steadily decreases and reaches a minimum of 32.7 GPa for the sample “WSEP-30”.

Furthermore, the depth of penetration of pressurized water and freezing and thawing resistance in the presence of salt are features of concrete with reference to its durability. Both types of testing showed no significant differences in results, indicating that the replacement of cement with waste sludge WSEP did not negatively affect the durability of concrete. The penetration depth of pressurized water ranges from 12 to 19 mm. The upper limit for the water impermeability class V-III (highest class) is 20 mm, indicating that the tested concretes satisfy the specified condition. The amount of scaled material on the concrete surface due to freezing/thawing in the presence of salt ranges from 0.14 to 0.20 mg/mm^2^, while the upper limit for the resistance class XF4 (highest class) is 0.20 mg/mm^2^, indicating that the tested concrete satisfies the above condition. The rebound number determined by the sclerometer measures the surface hardness of concrete, based on which the compressive strength can be estimated. This method is also used in the research to prove the uniformity of concrete quality. According to the test results, it can be seen that the rebound number decreases with an increasing percentage of cement replacement with waste sludge WSEP, similar to the case of compressive strength. The rebound value declines slightly from 48.6, recorded for reference concrete, to 42.4 for concrete labeled “WSEP-30”. This test confirmed that the quality of concrete, above all the compressive strength, declines slightly with an increasing content of waste sludge WSEP. The ultrasonic pulse velocity depends primarily on the bulk density and porosity of the concrete and the degree of achieved hydration. The obtained test results indicated that the ultrasonic pulse velocity through concrete slightly decreases with increased waste sludge WSEP.

Finally, replacing cement with waste sludge WSEP in raw form in concrete production can be an alternative to the existing production technologies, eliminating some of the expensive and energy-intensive methodologies of concrete production or sludge disposal. More importantly, for the first time, it was demonstrated that waste sludge generated in the water treatment processes of heating device production as environmentally harmful waste material could be transformed into a safe and stable product for the construction industry.

### 4.4. Parameters of Self-Compacting Concrete (SSC) Produced with Waste Sludge WSLP as a Powder Mineral Additive

It was established that the waste sludge WSLP does not possess pozzolanic activity and does not meet the criteria for tests of the activity index according to Standard EN 450-1 (Table 6). However, due to its chemical inertness and fineness, sludge WSLP was further investigated as a powdered mineral admixture for stone filler in self-compacting concrete (SCC). Since the parameters obtained during the testing of pozzolanic activity and the impact of cement replacement by WSLP on the properties of cement paste for all batches were continuous (Table 6), the analysis of the results obtained for SCC mixtures was presented based on the test on the sludge WSLP generated in November 2019, as the most representative. Namely, powdered mineral admixtures are a mandatory component of self-compacting concrete, and depending on the class of concrete, it can be dosed in the amount of 120–250 kg/m^3^ of concrete. Self-compacting concretes are new-generation concretes that are becoming more widely used in construction. The essential characteristic of these concretes is self-compacting, i.e., compacting without additional mounting means such as pervibrators and vibrotables. For the research, two SCC mixtures were made, the first (reference mixture; label E-SCC) with limestone filler as a mineral powder admixture and the second in which waste sludge replaced the limestone filler entirely (label WSLP-SCC). Table 9 shows the testing results on fresh and hardened concrete.

**Figure 15:**
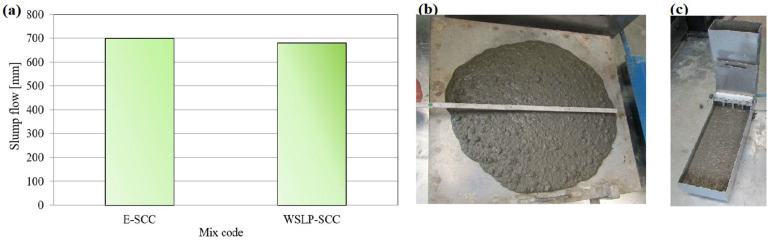
(**a**) SCC concrete slump measuring results; visual display of testing SCC concrete: (**b**) slump flow test and (**c**) L–box test.

Both SCC concrete mixtures were designed to have approximately the same slump (about 700 mm) when testing the fluidity of concrete (Figure 15a,b), which was achieved by applying superplasticizers. To achieve the target fluidity of SCC concrete mixed with waste sludge WSLP, 90% more superplasticizers were required than in the case of the reference concrete. This is a consequence of the observed porosity of the waste sludge WSLP (Table 4) and the tendency for water retention (Table 6), which reduces the amount of free water and, therefore, the fluidity in the concrete mix. The T_500_ test shows the viscosity of the concrete mix and represents the time over which concrete reaches a 500 mm slump when tested for fluidity. Based on the test results, the concrete mixture with the addition of waste sludge WSLP has twice the value of T500 compared to the reference concrete, indicating the increased viscosity of this mixture.

Passing ability was determined using L–box (Figure 15c). The test results indicate that the “WSLP-SCC” mixture has a lower ability to pass obstacles than the reference concrete “E-SCC”, which indicates that replacing the stone filler with waste sludge WSLP harms this SCC property. Testing segregation resistance is expressed as a percentage of the amount of concrete that passed the 5 mm opening sieve concerning the total mass. Based on the test results, it can be concluded that the mixture in which the stone filler is replaced using sample WSLP has higher segregation resistance than the reference concrete.

Replacement of the stone filler with waste sludge WSLP reduced the bulk density in fresh and solid mortar states. The difference is 2.5% due to the difference in the specific masses of waste sludge WSLP and the stone filler. Replacing the stone filler with waste sludge WSLP does not significantly impact the content of the entrained air in fresh concrete. The reference mixture “E-SCC” had a higher percentage of entrained air (2.2%) than “WSLP-SCC” (1.8%).

**Figure 16:**
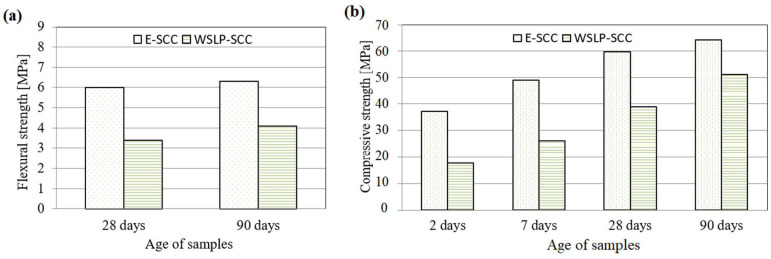
(**a**) Flexural strength of SCC concrete; (**b**) compressive strength of SCC concrete.

The flexural strength test was performed over 28 and 90 days (Figure 16a). Replacing the stone filler with waste sludge, WSLP contributed to a significant decrease in the flexural strength of self-reinforcing concrete (35% at 90 days). The most significant concrete characteristic, compressive strength, is determined for test specimens aged 2, 7, 28, and 90 days (Figure 16b). The compressive strengths of SCC mixtures with the incorporation of waste sludge WSLP are low at an early age due to the retarding effect of waste sludge WSLP on the cement hydration process. At higher ages of concrete, a more significant increase in the strength of “WSLP-SCC” was recorded, but it was lower than the strength of the standard “E-SCC” (at 90 days, the tensile strength of “WSLP-SCC” was 20% less than the “E-SCC”). Replacement of the stone filler with waste sludge WSLP contributed to the reduction in tensile splitting strength and the elasticity secant modulus of self-compacting concrete. This reduction is 32% for tensile strength and 11% for the secant modulus of elasticity.

The depth of penetration of pressurized water and resistance to freezing/thawing in the presence of salt determine concrete’s durability. The penetration depth of pressurized water for “E-SCC” and “WSLP-SCC” mixtures is 6 mm and 10 mm, respectively. The upper limit for the water-impermeability class V-III (highest class) is 20 mm, indicating that the tested concretes satisfy the above condition. The amount of scaling material on the concrete surface due to freezing/thawing in the presence of salt for the “E-SCC” and “WSLP-SCC” mixtures is 0.14 and 0.26 mg/mm^2^, respectively. The upper limit for the XF4 resistance class (highest class) is 0.20 mg/mm^2^, and for the XF2 resistance class, 0.30 mg/mm^2^, indicating that replacement of the stone filler with waste sludge WSLP reduces the freeze/thaw resistance class in the presence of salt. The rebound number determined by the sclerometer measures the surface hardness of the concrete, upon which the compressive strength can be estimated. This method is also used in research to prove the uniformity of concrete quality. The test results showed that the rebound number decreased due to the replacement of stone filler with waste sludge WSLP, similar to the case of compressive strength. The ultrasonic pulse velocity depends primarily on the bulk density and porosity of the concrete and the degree of hydration achieved. The test results showed that the speed of ultrasonic pulse velocity through SCC with waste sludge WSLP is slightly lower than that of the reference concrete.

Based on the results of testing, it can be concluded that waste sludge WSLP can be used as a substitute for a limestone filler (or any other mineral powder admixture) in the production of SSC up to 100%, with a maximum quantity of up to 100 kg per m^3^. However, due to its higher porosity and water absorption, it is necessary to increase the content of the chemical superplasticizer-type admixture to achieve optimal fluidity of the concrete. The addition of waste sludge WSLP delays the cement setting time and requires slightly longer concrete curing in the formwork. However, although the application of waste sludge WSLP in SCC concrete as a replacement for limestone filler negatively affects the mechanical properties, the durability of the concrete is not impaired. Moreover, considering that waste sludge WSLP might solve the disposal issues and, at the same time, can be used as a substitute for mineral powder admixture, it is justified to use it in the production of SCC. The use of waste WSLP sludge directly in the production of SCC concrete, without prior processing or expensive treatments in different incinerators, gives the possibility of new benefits and savings, which makes this method of finding particularly attractive.

## 5. Conclusions with Recommendations

The present research results showed that the waste sludges WSEP and WSLP from the wastewater treatment plants successfully found their usefulness and value in new products. It has been proven that both waste sludges have significant utilization potential and fully meet the necessary criteria.

Based on the results of waste sludge WSEP pozzolanic activity, as well as on its influence on the properties of cement paste, mortar, and concrete, a conclusion can be made that waste sludge WSEP showed pozzolanic properties and meets the criteria prescribed by the Standard EN 450-1 for application as admixture type II for concrete production according to EN 206-1 and can be used for cement substitution in mortar and concrete production processes. Investigations of the physicochemical and mechanical properties of waste sludge WSEP have justified its application as a replacement material for cement in mortar and concrete. However, a maximum cement replacement of up to 20% is recommended since the physical and mechanical properties are not significantly impaired compared to the etalon mortars and concrete prepared without waste additions.

Waste sludge WSLP does not possess pozzolanic properties and does not satisfy the criteria prescribed by Standard EN 450-1, which is related to the possibility of material use as an admixture type II for the production of concrete following Standard EN 206-1; therefore, it cannot be used as a replacement for cement in mortar and concrete. However, due to its chemical inertness and fineness, as well as extended setting time, WSLP can be used as a replacement for stone filler (or other powdered mineral admixture such as fly ash, red sludge, milled granular slurry from blast furnaces and silicate dust) in the production of self-compacting concrete (SCC). Investigations of the physicochemical properties have shown that applying WSLP in SCC causes a slight decrease in mechanical properties but does not reduce concrete durability. Waste sludge WSLP can replace powdered mineral admixture in producing up to 100% SSC, with a maximum quantity of up to 100 kg/m^3^.

Production of mortars, concrete, and SCC with investigated waste sludges WSEP and WSLP incorporated in their composition exhibited good and acceptable features and met all legislative requirements for fabrication, with great potential for realization on an industrial scale. Even more, by applying a globally accepted so-called zero waste strategy where each industrial plant strives to clean production by following green economy norms and where all types of waste from the production are maximally reused, the possibility of minimizing quantities of waste and reducing environmental pollution levels are provided. Moreover, such a conceptual framework may consequently lead to significant economic benefits. Finally, the use of sludge directly in the construction industry, without prior processing or expensive treatments in different incinerators, gives the possibility of new environmental benefits and economic savings, which makes these methods of finding the utility value of the generated sludges particularly attractive and a significant challenge for future feasible technologies.

## Figures and Tables

**Table 1:** Literature overview on utilizing different types of waste sludges in cement and concrete production.

Type of Waste Sludge	Tests Conducted	Waste Share	Ref. No.
Sewage and PAC Sludge	Compressive Strength, Density.	10% of sewage sludge and 5% of PAC sludge as a substitution for cement	[9]
Sewage Sludge	Slump Test, Compressive Strength, Dry Density.	5% of the cement weight in the concrete mix	[21]
Acid-washed Sewage Sludge	Compressive Strength, Evaluation of Hydraulic and Pozzolanic Properties.	10% of sludge as a replacement for cement in concrete and mortar	[22]
Alum Sludge	Slump test, Mechanical and Durability Properties (Compressive and Flexural Strength), Water Absorption, Gas Permeability, Chloride Penetration, Leaching Concentration of Aluminum.	4% or 8% replacement level of cement by alum sludge	[23]
Physicochemical Wastewater Sludge (from Marble Processing Plant)	Slump test, Compressive Strength, the Freeze/Thaw Resistance, Water Absorption, Capillary Suction, Porosity.	15% admixing level of sludge by weight of cement	[24]
Municipal Wastewater Treatment Sludge	Compressive and Flexural Tensile Strength, Density and Total Porosity, Leachability of Heavy Metals.	Up to 30% of waste sludge replacement for the cement.	[25]
Electroplating Industry Sludge	Compressive Strength	Up to 15% replacement of sand in cement mortar. Up to 20% replacement of sand in cement concrete.	[26]

**Table 2:** Content of experimentally determined heavy metals and calculated amounts of oxides in waste sludge WSEP in batches generated in October (2019), November (2019), December (2019), and January (2020).

Content, mg/kg						
Br.	Parameter	October 2019 [56]	November 2019	December 2019	January 2019	Reference Values
1.	Mo	7.5	7.5	9.5	6.5	10 * 30 **
2.	Hg	<0.15	<0.15	<0.15	<0.15	0.2 * 2.0 **
3.	Sb	85.5	86.5	81.5	79.5	0.7 * 5.0 **
4.	Se	2.5	3.5	2.5	35	0.5 * 7.0 **
5.	Sr	130.0	135.0	132.5	131.0	/*/**
6.	Ba	23.5	22.5	23.0	33.5	100 * 300 **
7.	Ca	8000.0	8002.0	8081.0	8002.0	/*/**
8.	Mg	430.0	431.0	433.0	438.0	/*/**
9.	Ti	1850	1840	1851	1860	/*/**
10.	V	14.5	12.0	13.0	13.5	/*/**
11.	Mn	4855.0	4859.0	4850.0	4812.0	/*/**
12.	Fe	13,250.0	13,270.0	13,324.0	13,257.0	/*/**
13.	Co	2330.0	2382.0	2334.0	2339.0	/*/**
14.	Cu	2050.0	2048.0	2055.0	2040.0	50 * 100 **
15.	Zn	175.5	179.5	178.5	174.0	50 * 200 **
16.	Ni	186.0	173.0	188.0	194.0	10 * 40 **
17.	Cd	6.0	6.0	6.5	6.0	1 * 5.0 **
18.	Al	1315.0	1313.0	1358.0	1312.0	/*/**
19.	Pb	37.0	37.0	35.0	38.0	10 * 50 **
20.	As	<0.5	<0.5	<0.5	<0.5	2 * 25 **
21.	Be	<0.05	<0.05	<0.05	<0.05	/*/**
22.	Cr	22.0	28.0	23.0	23.0	10 * 70 **
23.	Tl	2.5	2.5	2.5	2.5	/*/**
24.	Sn	<1.0	<1.0	<1.0	<1.0	/*/**
25.	Si	50,409.0	48,888.0	48,384.0	47,856.0	/*/**
26.	MoO_3_	11.25	11.25	14.25	9.75	/*/**
27.	HgO	<0.15	<0.15	<0.15	<0.15	/*/**
28.	Sb_2_O_3_	204.69	207.09	195.12	190.33	/*/**
29.	SeO_2_	3.51	4.92	3.51	49.18	/*/**
30.	SrO	153.73	159.64	156.69	154.91	/*/**
31.	BaO	0.00	25.12	25.68	37.40	/*/**
32.	CaO	11,198.80	11,201.60	11,312.19	11,201.60	/*/**
33.	MgO	713.02	714.68	718.00	726.29	/*/**
34.	TiO_2_	3093.23	3076.51	3094.90	3109.95	/*/**
35.	V_2_O_5_	51.76	42.84	46.41	48.19	/*/**
36.	MnO	6268.38	6273.54	6261.92	6212.86	/*/**
37.	Fe_2_O_3_	37,883.38	37,940.56	38,094.95	37,903.39	/*/**
38.	Co_3_O_4_	9608.37	9822.81	9624.87	9645.49	/*/**
39.	CuO	2565.94	2563.43	2572.19	2553.42	/*/**
40.	ZnO	218.43	223.40	222.16	216.56	/*/**
41.	NiO	236.48	219.95	239.02	246.65	/*/**
42.	CdO	6.85	6.85	7.42	6.85	/*/**
43.	Al_2_O_3_	4885.05	4877.62	5044.79	4873.91	/*/**
44.	PbO	39.86	39.86	37.70	40.93	/*/**
45.	As_2_O_3_	<0.5	<0.5	<0.5	<0.5	/*/**
46.	BeO	<0.05	<0.05	<0.05	<0.05	/*/**
47.	Cr_2_O_3_	64.30	81.84	67.22	67.22	/*/**
48.	Tl_2_O_3_	5.59	5.59	5.59	5.59	/*/**
49.	SnO_2_	<1.0	<1.0	<1.0	<1.0	/*/**
50.	SiO_2_	108,012.0	104,761.0	103,685.0	102,654.8	/*/**

According to “Rulebook on the categories, testing and classification of waste (Sl. glasnik 56/2010, 93/2019, 39/2021)”: * values valid for the disposal of non-reactive hazardous waste at non-hazardous landfills in cassettes not intended for disposing of biodegradable waste; ** values valid for the disposal at a hazardous waste landfill.

**Table 3:** Content of experimentally determined heavy metals and calculated amounts of oxides in waste sludge WSLP in batches generated in October (2019), November (2019), December (2019), and January (2020).

Content, mg/kg						
Br.	Parameter	October 2019 [56]	November 2019	December 2019	January 2019	Reference Values
1.	Mo	0.15	0.15	0.15	0.15	10 * 30 **
2.	Hg	<0.15	<0.15	<0.15	<0.15	0.2 * 2.0 **
3.	Sb	0.85	0.85	0.75	0.95	0.7 * 5.0 **
4.	Se	<0.2	<0.2	<0.3	<0.2	0.5 * 7.0 **
5.	Sr	42.0	41.0	44.0	39.0	/*/**
6.	Ba	52.0	51.0	52.0	51.0	100 * 300 **
7.	Ca	46,700.0	46,708.0	46,735.0	46,778.0	/*/**
8.	Mg	2920.0	2925.0	2944.0	2933.0	/*/**
9.	Ti	56.0	53.0	51.0	55.0	/*/**
10.	V	5.5	65	5.0	5.0	/*/**
11.	Mn	600.0	609.0	620.0	602.0	/*/**
12.	Fe	18,750.0	18,755.0	18,745.0	18,752.0	/*/**
13.	Co	3.5	4.5	4.5	0.5	/*/**
14.	Cu	20.5	22.5	22.5	19.5	50 * 100 **
15.	Zn	37.0	32.0	33.0	35.0	50 * 200 **
16.	Ni	8.0	9.0	9.0	8.0	10 * 40 **
17.	Cd	2.5	2.5	3.5	2.5	1 * 5.0 **
18.	Al	2045.0	2042.0	2023.0	2075.0	/*/**
19.	Pb	10.0	11.0	12.0	10.0	10 * 50 **
20.	As	2.5	2.5	2.5	2.5	2 * 25 **
21.	Be	<0.05	<0.05	<0.05	<0.05	/*/**
22.	Cr	8.0	9.0	7.0	5.0	10 * 70 **
23.	Tl	<0.5	<0.5	<0.5	<0.5	/*/**
24.	Sn	<1.0	<1.0	<1.0	<1.0	/*/**
25.	Si	9193.3	10,112.7	9653.0	9009.5	/*/**
26.	MoO_3_	0.23	0.23	0.23	0.23	/*/**
27.	HgO	<0.15	<0.15	<0.15	<0.15	/*/**
28.	Sb_2_O_3_	2.03	2.03	1.80	2.27	/*/**
29.	SeO_2_	<0.2	<0.2	<0.3	<0.2	/*/**
30.	SrO	49.67	48.48	52.03	46.12	/*/**
31.	BaO	58.06	56.94	58.06	56.94	/*/**
32.	CaO	65,373.00	65,384.19	65,421.99	65,482.18	/*/**
33.	MgO	4841.91	4850.20	4881.71	4863.47	/*/**
34.	TiO_2_	93.63	88.62	85.27	91.96	/*/**
35.	V_2_O_5_	19.63	232.04	17.85	17.85	/*/**
36.	MnO	774.67	786.29	800.49	777.25	/*/**
37.	Fe_2_O_3_	53,608.55	53,622.85	53,594.25	53,614.27	/*/**
38.	Co_3_O_4_	14.43	18.56	18.56	2.06	/*/**
39.	CuO	25.66	28.16	28.16	24.41	/*/**
40.	ZnO	46.05	39.83	41.07	43.56	/*/**
41.	NiO	10.17	11.44	11.44	10.17	/*/**
42.	CdO	2.86	2.86	4.00	2.86	/*/**
43.	Al_2_O_3_	7596.90	7585.76	7515.18	7708.35	/*/**
44.	PbO	10.77	11.85	12.93	10.77	/*/**
45.	As_2_O_3_	6.60	6.60	6.60	6.60	/*/**
46.	BeO	<0.05	<0.05	<0.05	<0.05	/*/**
47.	Cr_2_O_3_	23.38	26.30	20.46		/*/**
48.	Tl_2_O_3_	<0.5	<0.5	<0.5	<0.5	/*/**
49.	SnO_2_	<1.0	<1.0	<1.0	<1.0	/*/**
50.	SiO_2_	19,722.0	21,673.0	20,685.2	19,306.6	/*/**

According to “Rulebook on the categories, testing and classification of waste (Sl. glasnik 56/2010, 93/2019, 39/2021)”: * values valid for the disposal of non-reactive hazardous waste at non-hazardous landfills in cassettes not intended for disposing of biodegradable waste; ** values valid for the disposal at a hazardous waste landfill.

**Table 4:** Porosity parameters of waste sludges WSEP and WSLP generated in October, November, December 2019, and January 2020.

Batch				October 2019					November 2019			
Sample	*S_p_*_,_ m^2^/g	*V_total_*, cm^3^/g	*V_meso_*, cm^3^/g	*V_micro_*, cm^3^/g	*D_sr_*, nm	*D_max_*, nm	*S_p_*_,_ m^2^/g	*V_total_*, cm^3^/g	*V_meso_*, cm^3^/g	*V_micro_*, cm^3^/g	*D_sr_*, nm	*D_max_*, nm
WSEP	60.7	0.1230	0.1152	0.0156	7.43	2.86	59.9	0.1301	0.1092	0.0209	8.02	2.79
WSLP	29.7	0.1413	0.1374	0.0069	14.06	3.62	33.6	0.1422	0.1279	0.0143	12.12	3.29
Batch				December 2019					January 2020			
Sample	*S_p_*_,_ m^2^/g	*V_total_*, cm^3^/g	*V_meso_*, cm^3^/g	*V_micro_*, cm^3^/g	*D_sr_*, nm	*D_max_*, nm	*S_p_*_,_ m^2^/g	*V_total_*, cm^3^/g	*V_meso_*, cm^3^/g	*V_micro_*, cm^3^/g	*D_sr_*, nm	*D_max_*, nm
WSEP	23.2	0.1230	0.1152	0.0156	8.01	2.87	18.06	0.0786	0.0757	0.0046	13.90	2.82
WSLP	128.3	0.1411	0.1362	0.0079	12.27	3.33	168.5	0.4920	0.4935	0.0545	8.55	3.74

**Table 5:** Results of pozzolanic activity and parameters of cement paste with the addition of the waste sludge WSEP.

	Class of Pozzolanic Materials	Activity Index	Water Requirement	Standard Consistence	Initial Setting Time Final Setting Time	Soundness
Standard	SRPS B.C1.018	EN 450-1 (25% Cement Replacement)	EN 450-1 Annex B	EN 196-3 (25% Cement Replacement)	EN 196-3 (25% Cement Replacement)	EN 196-3 (30% Cement Replacement)
**November 2019**	Flexural strength— 2.23 MPa > 2.0 MPa (Requirements SRPS B.C1.018 p. 5.3 for class 5)	28 days—75.14% > 75% (Requirements EN 450-1 p. 5.3.2)	104% < 95% (Requirements EN 450-1 p. 5.3.6)	33.5% (100% cement—29.0%) ((Not meet the condition for standard consistence)	315 min < 2 × 100% cement (100% cement—115 min) (Requirements EN 450-1 p. 5.3.5)	1.0 mm (100% cement—1.0 mm) < 10 mm (Requirements EN 450-1 p. 5.3.3)
	Compressive strength— 7.90 MPa > 5.0 MPa (Requirements SRPS B.C1.018 p. 5.3 for class 5)	90 days—86.0 > 85% (Requirements EN 450-1 p. 5.3.2)			340 min (100% cement—150 min) (Not meet the condition for final setting time)	
**Criteria**	Satisfied (Class 5 of pozzolanic materials)	Satisfied	Not satisfied	/	Not satisfied	Satisfied
	Satisfied (Class 5 of pozzolanic materials)	Satisfied			/	

**Table 6:** Results of pozzolanic activity and parameters of cement paste with the addition of the waste sludge WSLP.

	Class of Pozzolanic Materials	Activity Index	Water Requirement	Standard Consistence	Initial Setting Time Final Setting Time	Soundness
Standard	SRPS B.C1.018	EN 450-1 (25% Cement Replacement)	EN 450-1 Annex B	EN 196-3 (25% Cement Replacement)	EN 196-3 (25% Cement Replacement)	EN 196-3 (30% Cement Replacement)
**November 2019**	Flexural strength— 0.20 MPa > 2.0 MPa (Requirements SRPS B.C1.018 p. 5.3 for class 5)	28 days—57.24% > 75% (Requirements EN 450-1 p. 5.3.2)	135% < 95% (Requirements EN 450-1 p. 5.3.6)	43.0% (100% cement—29.0%) ((Not meet the condition for standard consistence)	440 min < 2 × 100% cement (100% cement—115 min) (Requirements EN 450-1 p. 5.3.5)	3.0 mm (100% cement—1.0 mm) < 10 mm (Requirements EN 450-1 p. 5.3.3)
	Compressive strength— 0.62 MPa > 5.0 MPa (Requirements SRPS B.C1.018 p. 5.3 for class 5)	90 days—61.23 > 85% (Requirements EN 450-1 p. 5.3.2)			500 min (100% cement—150 min) (Not meet the condition for final setting time)	
**Criteria**	Not satisfied (without pozzolanic activity)	Not satisfied	Not satisfied	/	Not satisfied	Satisfied
	Not satisfied (without pozzolanic activity	Not satisfied			/	

**Table 7:** Testing results of mortar mixes based on Portland cement, natural sand, and added waste sludge WSEP.

No.	Property	Standard	Designation	Results	
1.	Consistency—by flow table	EN 1015-3	E	135 mm	
			WSEP-7.5	130 mm	
			WSEP-15	127 mm	
			WSEP-22.5	121 mm	
			WSEP-30	116 mm	
2.	Bulk density of fresh mortar	EN 1015-6	E	2299 kg/m^3^	
			WSEP-7.5	2290 kg/m^3^	
			WSEP-15	2285 kg/m^3^	
			WSEP-22.5	2360 kg/m^3^	
			WSEP-30	2254 kg/m^3^	
3.	Dry bulk density of hardened mortar	EN 1015-10	E	2294 kg/m^3^	
			WSEP-7.5	2287 kg/m^3^	
			WSEP-15	2280 kg/m^3^	
			WSEP-22.5	2357 kg/m^3^	
			WSEP-30	2250 kg/m^3^	
4.	Water absorption	EN 13755	E	7.54%	
			WSEP-7.5	7.80%	
			WSEP-15	8.26%	
			WSEP-22.5	8.79%	
			WSEP-30	9.27%	
5.	Water absorption	EN 1015-18		Results are given in Figure 7.	
6.	Shrinkage	SRPS B.C8.029:1979 (ASTM C 596)		Results are given in Figure 8.	
7.	Adhesion of a concrete substrate	EN 1015-12	E	2.0 N/mm^2^	
			WSEP-7.5	1.9 N/mm^2^	
			WSEP-15	1.8 N/mm^2^	
			WSEP-22.5	1.5 N/mm^2^	
			WSEP-30	1.4 N/mm^2^	
8.	Leaching test	EN 12457-2	Element	Concentration (mg/dm^3^)	MDK
			Mo	<0.05	-
			Hg	<0.001	<0.001 *
			Sb	0.49	-
			Se	<0.05	-
			Sr	1.794	-
			Ba	0.549	-
			Ca	1.588(100×)	-
			Mg	0.063	-
			Ti	0.40	-
			V	<0.010	-
			Mn	<0.05	-
			Fe	<0.05	-
			Co	<0.05	-
			Cu	<0.05	<0.1
			Zn	<0.05	<1.0
			Ni	0.02	<0.1 *
			Cd	<0.01	<0.01 *
			Al	47.50	-
			Pb	<0.05	<0.1 *
			As	<0.05	<0.05 *
			Be	<0.05	-
			Cr	<0.05	<0.5 *
			Tl	<0.05	-
			Sn	<0.05	-
			Si	213.54	-

* Rulebook on permitted quantities of hazardous and harmful substances in soil and irrigation water and their testing methods (Sl. glasnik 23/1994-553).

**Table 8:** Testing concrete results based on Portland cement, natural sand, large crushed aggregate, and added waste sludge WSEP.

Testing of the Mix-Design of the Concrete Based on Portland Cement, Natural Sand, Coarse Crushed Aggregate, and the Addition of Waste Sludge WSEP (November 2019)				
1.	Consistency—slump test; density of fresh concrete	EN 12350-2	E	200 mm
			WSEP-10	60 mm
			WSEP-20	10 mm
			WSEP-30	0 mm
2.	Density of fresh concrete	EN 12350-6	E	2466 kg/m^3^
			WSEP-10	2479 kg/m^3^
			WSEP-20	2485 kg/m^3^
			WSEP-30	2470 kg/m^3^
3.	Air content in fresh concrete	EN 12350-7	E	2.6%
			WSEP-10	2.3%
			WSEP-20	2.1%
			WSEP-30	1.8%
4.	Density of hardened concrete (water-saturated)	EN 12390-7	E	2455 kg/m^3^
			WSEP-10	2471 kg/m^3^
			WSEP-20	2480 kg/m^3^
			WSEP-30	2465 kg/m^3^
5.	Flexural strength	EN 12390-5	E	7.0 MPa (28 days), 7.4 MPa (90 days)
			WSEP-10	6.0 MPa (28 days), 6.6 MPa (90 days)
			WSEP-20	5.6 MPa (28 days), 6.3 MPa (90 days)
			WSEP-30	5.3 MPa (28 days), 6.0 MPa (90 days)
6.	Compressive strength	EN 12390-3	E	38.8 MPa (2 days), 56.3 MPa (7 days), 66.5 MPa (28 days), 73.6 MPa (90 days)
			WSEP-10	36.2 MPa (2 days), 56.5 MPa (7 days), 64.5 MPa (28 days), 71.3 MPa (90 days)
			WSEP-20	34.4 MPa (2 days), 49.6 MPa (7 days), 56.9 MPa (28 days), 64.4 MPa (90 days)
			WSEP-30	25.8 MPa (2 days), 44.1 MPa (7 days), 53.7 MPa (28 days), 60.2 MPa (90 days)
7.	Tensile splitting strength	EN 12390-6	E	3.9 MPa (28 days)
			WSEP-10	3.6 MPa (28 days)
			WSEP-20	3.3 MPa (28 days)
			WSEP-30	3.2 MPa (28 days)
8.	Secant modulus of elasticity	EN 12390-13	E	33.0 GPa (28 days)
			WSEP-10	33.3 GPa (28 days)
			WSEP-20	33.1 GPa (28 days)
			WSEP-30	32.7 GPa (28 days)
9.	Depth of penetration of water under pressure	EN 12390-8	E	12 mm
			WSEP-10	14 mm
			WSEP-20	17 mm
			WSEP-30	19 mm
10.	Freeze–thaw resistance with de-icing salts—scaling	CEN-TS_12390-9	E	0.14 mg/mm^2^
			WSEP-10	0.15 mg/mm^2^
			WSEP-20	0.18 mg/mm^2^
			WSEP-30	0.20 mg/mm^2^
11.	Determination of rebound number	EN 12504-2	E	48.6
			WSEP-10	47.0
			WSEP-20	45.7
			WSEP-30	42.4
12.	Determination of ultrasonic pulse velocity	EN 12504-4	E	5.21 km/s
			WSEP-10	5.18 km/s
			WSEP-20	5.15 km/s
			WSEP-30	5.13 km/s

**Table 9:** Results of testing of SCC produced with waste sludge WSLP as mineral admixture.

Testing of SCC Produced with Waste Sludge WSLP as Mineral Admixture (November 2019)				
1.	Consistency—slump flow test	EN 12350-8	E-SCC	700 mm
			WSLP-SCC	680 mm
2.	T_500_ test	EN 12350-8	E-SCC	4.2 s
			WSLP-SCC	9.1 s
3.	L-box passing ratio (H_2_/H_1_)	EN 12350-10	E-SCC	0.94 (mm/mm)
			WSLP-SCC	0.81 (mm/mm)
4.	Testing segregation using sieves	EN 12350-11	E-SCC	12.6%
			WSLP-SCC	3.1%
5.	Density of fresh concrete	EN 12350-6	E-SCC	2460 kg/m^3^
			WSLP-SCC	2400 kg/m^3^
6.	Air content in fresh concrete	EN 12350-7	E-SCC	2.2%
			WSLP-SCC	1.8%
7.	Density of hardened concrete (water-saturated)	EN 12390-7	E-SCC	2457 kg/m^3^
			WSLP-SCC	2392 kg/m^3^
8.	Flexural strength	EN 12390-5	E-SCC	6.0 MPa (28 days), 6.3 MPa (90 days)
			WSLP-SCC	3.4 MPa (28 days), 4.1 MPa (90 days)
9.	Compressive strength	EN 12390-3	E-SCC	37.2 MPa (2 days), 49.1 MPa (7 days), 59.7 MPa (28 days), 64.2 MPa (90 days)
			WSLP-SCC	17.6 MPa (2 days), 26.2 MPa (7 days), 38.8 MPa (28 days), 51.1 MPa (90 days)
10.	Tensile splitting strength	EN 12390-6	E-SCC	4.1 MPa (28 days)
			WSLP-SCC	2.8 MPa (28 days)
11.	Secant modulus of elasticity	EN 12390-13	E-SCC	29.5 GPa (28 days)
			WSLP-SCC	26.3 GPa (28 days)
12.	Depth of penetration of water under pressure	EN 12390-8	E-SCC	6 mm
			WSLP-SCC	10 mm
13.	Freeze–thaw resistance with de-icing salts—scaling	CEN-TS_12390-9	E-SCC	0.14 kg/m^2^
			WSLP-SCC	0.26 kg/m^2^
14.	Determination of rebound number	EN 12504-2	E-SCC	45.6
			WSLP-SCC	42.1
15.	Determination of ultrasonic pulse velocity	EN 12504-4	E-SCC	4.87 km/s
			WSLP-SCC	4.70 km/s

## Data Availability

The data presented in this study are available on request from the corresponding author. The data are not publicly available due to privacy.

## Supplementary Materials

The following supporting information can be downloaded at: https://www.mdpi.com/article/10.3390/ma17051089/s1.
