# Supplementary material for: Sustainable Application of Waste Sludges from the Wastewater Treatment Plant Generated during the Production of Heating Devices in the Construction Industry
Source: Materials (Basel). 2024 Feb 27;17(5):1089. doi: 10.3390/ma17051089 (PMC10935120; doi:10.3390/ma17051089)
Supplement: Supplementary file 1 [file materials-17-01089-s001.zip › Supplementary Material S1.pdf]

Supplementary Materials S1

2.3.1. Investigation of the pozzolanic activity and parameters of the cement paste with addition of waste sludges

In order to determine the potential use of waste sludges WSEP and WSLP, which were generated in the October, November, December 2019 and January 2020 as the type II admixtures in making of concrete in accordance with the standard EN 206, the following parameters were investigated with implementation of standard methods.

1. **Class of pozzolanic materials, SRPS B.C1.018**, is determined based on the mechanical strengths of mortars. The investigated pozzolanic material should be finer than 0.063 mm and dried at the temperature of 98 ºC. For making of the mortar, 1350 g of standard three-fraction sand, 300 g of pozzolanic material, 150 g of standard hydrated lime and 270 cm^3 of water were mixed. Mechanical strengths are tested on specimens with dimensions 40 mm x 40 mm x 160 mm using hydraulic press machine. After preparing, the specimens were hermetically sealed in a tin box. After the initial 24 h spent in laboratory conditions, specimens were cured at the temperature of 55 °C for six days and then tested.
2. **Activity index, EN 450-1** is the ratio of compressive strength of the test mortar made with 75 % of cement and 25 % of pozzolanic material and of the reference mortar (made with 100 % of cement) whereby mortar samples are of the same age. Pozzolanic material should be finer than 0.063 mm and dried at the temperature of 98 ºC. For making of the mortar, 1350 g of standard three-fraction sand, 337 g of cement, 112 g of pozzolanic material and 225 g of water were used, while the reference mortar was made with the same quantity of standard sand and water, but with 450 g of cement i.e. without the pozzolanic material. Compressive strength was tested on the hydraulic press machine on the prism-shaped specimens with dimensions 40 x 40 x 160 mm at the age of 28 and 90 days.
3. **Water requirement, EN 450-1 Annex B** represents the percentage ratio of the quantity of water required for making of the test mortar made with 70 % of cement and 30 % of pozzolanic material and reference mortar made with 100 % of cement. For making of the reference mortar 1350 g of three-fraction standard sand, 450 g of cement and 225 g of water were mixed, while for the making of the test mortar, 1350 g of three-fraction standard sand, 315 g of cement and 135 g of pozzolanic material were used, as well as the corresponding quantity of water, required in order to provide the results of slump tests on the flow table (according to EN 1015-3) of test and reference mortar, which do not differ more than ± 10 mm.
4. **Standard consistence, EN 196-3** represents the percentage ratio of the amount of water and amount of cement in the cement paste, which can meet the condition that the Tetmeyer probe of the Vicat apparatus passing through the cement paste that is poured in the testing ring, stops at 6±2 mm from the base glass panel. The test mixture is made with 75 % of cement and 25 % of pozzolanic material, i.e. 375 g of cement, 125 g of pozzolanic material and appropriate amount of water for fulfilling previously mentioned condition.
5. **Initial setting time and Final setting time, EN 196-3** are the parameters determined on the cement paste of standard consistency. The initial setting time is the time that has passed from the moment when water and binder were mixed to the moment when the needle of the Vicat apparatus stoped at 6±3 mm from the base glass panel. The final setting time is the time that has passed form the moment when water and binder were mixed to the moment when the needle of the Vicat apparatus penetrated no more then 0.5 mm into the cement paste.
6. **Soundness, EN 196-3** is also an important parametar determined on the cement paste of standard consistency. Le Chatelier rings filled with cement paste were used and cured for 24 h in a water bath, and then boiled for 2.5 h. The distance between the needles of Le Chatelier rings prior and after boiling was measured. Based on that value, the soundness was evaluated (the distance should not exceed 10 mm).

2.3.2. Preparing of the mortar mixtures and investigation of the parametars of mortars based on Portland cement, natural sand and the addition of waste sludge WSEP

For the purpose of standard parameters investigation, a total of five different mortar mixtures were prepared: an etalon mixture as reference mix-design (marked with E; Table S1) and four mixtures in which cement was partially replaced with sludge WSEP in the amount of 7.5 %, 15 %, 22.5 % and 30 % by weight (marked as WSEP-7.5, WSEP-15, WSEP-22.5, WSEP-30, respectively; Table S1).

Table S1. The mix-design of the mortar mixtures based on Portland cement, natural sand and added waste sludge WSEP generated in the October, November, December 2019 and January 2020.

(E)	WSEP-7.5	WSEP-15	WSEP-22.5	WSEP-30
Cement: 450 g<br>WSEP: /<br>River sand (0-2 mm): 1350 g<br>Water: 225 g<br>Superplasticizer: 1.0 g	Cement: 416.25 g<br>WSEP: 33.75 g<br>River sand (0-2 mm): 1350 g<br>Water: 225 g<br>Superplasticizer: 1.0 g	Cement: 382.5 g<br>WSEP: 67.5 g<br>River sand (0-2 mm): 1350 g<br>Water: 225 g<br>Superplasticizer: 1.0 g	Cement: 348.75 g<br>WSEP: 101.25 g<br>River sand (0-2 mm): 1350 g<br>Water: 225 g<br>Superplasticizer: 1.0 g	Cement: 315 g<br>WSEP: 135 g<br>River sand (0-2 mm): 1350 g<br>Water: 225 g<br>Superplasticizer: 1.0 g

Following parameters of mortar mixtures based on the Portland cement, natural sand and the admixture of waste sludge WSEP in the precisely defined percentage were determined with implementation of standard methods, as explained:

1. **Consistency – by flow table, EN 1015-3** was performed on the flow table. For the test, a metal conical mold was used, with the lower base diameter of 100 mm. The metal cone was set in the middle of the flow table and filled with fresh mortar in two layers, each layer being compacted using a wooden compactor. After that, the metal cone was removed, and the table was falling from a height of 1 cm 15 times in a period of 15 s. After that, mortar slump in two orthogonal directions was measured. The mean value represented the final mortar slump.
2. **Bulk density of fresh mortar, EN 1015-3** is determined in a 1 dm^3 cylindrical metal vessel with diameter of 125 mm. Mass of the empty vessel was measured initially, and then the vessel was filled with mortar, which was compacted using the vibrating table. After that, excess mortar was removed, upper layer of mortar was flattened, and mass of the full vessel was measured again. The ratio of the difference of masses of the full and empty vessel and vassels volume capacity represent the bulk density of freshly compacted mortar.
3. **Bulk density of hardened mortar, EN 1015-10** is determined on the specimens used for testing of mechanical strengths, i.e. on the prisms with dimensions 40×40×160 mm. The mass of each prism-shaped specimen was measured and divided with the volume calculated based on its dimensions. The ratio represented the bulk density of hardened mortar.
4. **Flexural strength, EN 196-1, (EN 1015-11)** was tested on the specimens in form of prisms with dimensions 40×40×160 mm. The tests were conducted on the hydraulic press UTEST UTCM - 6710 with a measuring range of the flexural force of 0-10 kN and accuracy class of 0.5 %. The flexural strength of mortar was calculated according to the following formula:
$$f_{s,i} = \frac{M_{max}}{W} = \frac{3}{2} \cdot \frac{F_s \cdot l_o}{b \cdot h^2} = \left[\frac{N}{mm^2}, MPa\right]$$
where: Fs is the flexural force leading to the prism failure in N; lo is axial distance between the supports in mm (100 mm); b, h are width and height of the cross section of the prism in mm; and $f_{s,i}$ is flexural strength in MPa.
5. **Compressive strength, EN 196-1 (EN 1015-11)** was tested on the halves of the prisms, which were obtained after testing of flexural strength. The test was conducted on the hydraulic press UTEST UTCM - 6710 of a measuring range of the compressive force of 0-250 kN and accuracy class of 0.5 %. Compressive strength of mortar was calculated according to the following formula:

$$f_{p,i} = \frac{F_p}{A} = \left[\frac{N}{mm^2}, MPa\right]$$

where Fp is the compressive force leading to the failure of halves of prisms in N; A is a surface acted upon by the force (40 x 40 = 1600 mm^2) and $f_{p,i}$ is compressive strength in MPa.

6. **Water absorption (at atmospheric pressure), EN 13755** was evaluated on the specimens in form of prisms with dimensions of 40×40×160 mm. Prisms were initially dried at the temperature of 70 ± 5 °C until the constant mass was achieved. Then the prisms were set on the linear supports in a deep vessel, where water was poured up to the half of the specimens height. After 60 minutes, water was poured up to ¾ of the specimens height, and after 120 minutes, the samples were totally immersed in water, so that the layer of water above the specimens was 25 mm high. After 48 h, specimens were removed from the water after, their mass was measured and afterwards they were placed again in water and after 24 h their mass was measured again as well. Namely, if the difference of mass values is less than 0,01g it can be considered that the specimens are saturated with water, otherwise the samples must be returned into the water and measured again. The percentage ratio of the difference between masses of a saturated and a dry sample, and the mass of the dry sample, represented the water absorption at atmospheric pressure.
7. **Water absorption (water absorption coefficient due to capillary action of hardened mortar), EN 1015-18)** was determined on the halves of the prisms with dimensions of 40×40×160 mm. Prisms were initially dried at the temperature of 60 ± 5 °C until the constant mass was reached (M0). Then the surface of the prism failure was set on the linear supports in a deep vessel, where water was poured in the way that the prism was immersed 5 to 10 mm in depth. Prior to the immersion, the mass of the dry sample was measured, and after the immersion, the mass of the sample was measured as well after 10 min (M1), 90 min (M2) and 24 h (M3). Water absorption coefficient due to capillary action was calculated according to the following formulas:
   C = 0.1 × (M2 - M1) [$kg/(m^2 \, min^{-0.5})$] – for coating mortars; C = 0.625 × (M3 - M0) [kg/m^2] – for repairing mortars.
8. **Drying shrinkage, SRPS B.C8.029:1979 (ASTM C 596)** of mortars was determined on the prism samples with dimensions 40×40×160 mm with reference marks at their ends allowing monitoring of variation in length. After preparing, mortar prisms were cured in a mold in a water bath for 24 h, after which they were removed from the mold and cured in water for the next 48 h. Baseline reading was conducted 72 h after preparation of the prisms, and then the readings were performed after 4, 7, 14, 21 and 28 days. The difference in readings represented variation in length of the tested samples, while shrinkage of cement mortar was expressed as the ratio of length variation and the initial length of the sample at the start of testing. Mortar shrinkage ε_{sm} was calculated according to the following equation:
   $$\varepsilon_{sm,i} = \frac{\Delta l_{sm}(t)}{l_{sm}} \quad [mm/m]$$
   where $\Delta l_{sm}(t)$ is the difference of the sample length at the moment t and baseline reading t_{72h} in mm, l_{sm} is the length of the mortar prism expressed in m and ε_{sm} is shrinkage of the mortar prism expressed in mm/m.
9. **Adhesion of a concrete substrate (Adhesive strength of hardened rendering and plastering mortars on substrates), EN 1015-12** is determined by so-called pull-off test. A layer of mortar 10±1 mm thick was applied on the concrete substrate. It was necessary to cure the mortar for 7 days in hermetically isolated environment at the temperature of 20± 2 °C. Afterwards, the mortar had to be additionally cured for 21 day in the air atmosphere at the temperature of 20 ± 2 °C and with air humidity of 65± 5 %. Mortar and concrete surface were cut to 2 mm of height using the cylindrical drill of 50 mm in diameter. Afterwards, a steel dolly of 50 mm in diameter was glued to the mortar using epoxy glue and the dolly was then pulled off using the pull-off tester. The pull off failure force was registered, as well as the location where the failure occurred. The adhesive strength of mortar to a concrete substrate was calculated according to the following formula:
   $$f_u = \frac{F}{A}$$
   where F is the failure force at pull-off and A is the surface area of the dolly.

10. **Leaching** was evaluated by **leaching tests**, performed according to Standard EN 12457-2 on mortar samples in which cement was replaced with waste material in quantities that were found to be the maximum allowed in previous tests. The samples were prepared so that the mass ratio of water: binder (cement and waste material): sand was 1 : 2 : 6. Tests were performed with representative samples of the waste materials in form of sludge from the filter press (waste sludge sample WSEP). Solid crushed mortar in amount of 100 g with a particle size of about 4 mm was prepared, in which cement was replaced for press sludge WSEP in the share of 30 %. The samples were washed with water in a total amount of 1 dm^3. After the test, the leachates were analyzed on the Analytic Jena Specol 1300 device, where the leached content of the following elements was determined: Mg, Fe, Co, Cu, Zn, Ni, Cd, Al, Pb, Cr, Sn, Si, Mo, Sb, Sr, Ca, Ti, Mn and V.

2.3.3. Preparing of the concrete mixtures and investigation of the parametars of comncrete based on Portland cement, natural sand, coarse crushed aggregate and the addition of waste sludge WSEP

For the purpose of standard parameters investigation, a total of four different concrete mixtures were prepared: an etalon mixture as reference mix-design (marked with E; Table S2) and three mixtures in which cement was partially replaced with sludge WSEP in the amount of 10 %, 20 % and 30 % by weight (marked as WSEP-19, WSEP-20 and WSEP-30, respectively; Table S2).

Table S2. The mix-design of the concrete based on Portland cement, natural sand, coarse crushed aggregate and added waste sludge WSEP generated in the November 2019.

E	WSEP-10	WSEP-20	WSEP-30
Cement 380 kg<br>WSEP: /<br>River sand (0-4 mm) – 43 % or 808 kg<br>Crushed aggregate (4/8 mm) – 20 % or 376 kg<br>Crushed aggregate (8/16 mm) – 37 % or 696 kg<br>Water 180.0 kg<br>Superplasticizer 3.04 kg (0.8 %)	Cement 342 kg<br>WSEP: 38 kg<br>River sand (0-4 mm) – 43 % or 808 kg<br>Crushed aggregate (4/8 mm) – 20 % or 376 kg<br>Crushed aggregate (8/16 mm) – 37 % or 696 kg<br>Water 180.0 kg<br>Superplasticizer 3.04 kg (0.8 %)	Cement 304 kg<br>WSEP: 76 kg<br>River sand (0-4 mm) – 43 % or 808 kg<br>Crushed aggregate (4/8 mm) – 20 % or 376 kg<br>Crushed aggregate (8/16 mm) – 37 % or 696 kg<br>Water 180.0 kg<br>Superplasticizer 3.04 kg (0.8 %)	Cement 266 kg<br>WSEP: 114 kg<br>River sand (0-4 mm) – 43 % or 808 kg<br>Crushed aggregate (4/8 mm) – 20 % or 376 kg<br>Crushed aggregate (8/16 mm) – 37 % or 696 kg<br>Water 180.0 kg<br>Superplasticizer 3.04 kg (0.8 %)

Following parameters of concrete mixtures based on the Portland cement, natural sand, coarse crushed aggregate and the addition of waste sludge WSEP in the precisely defined percentage were determined with implementation of standard methods, as explained:

1. **Consistency – slump test, EN 12350-2** was determined using the Abrams cone slump method. The Abrams cone with hight of 30 cm and diameter of 20 cm at the base was set on a flat surface and filled with fresh concrete in three layers, whereby each layer was compacted using a metal rod. Afterwards, within 5s the cone was lifted vertically and placed on the same surface where concrete was poured on. A metal ruler was placed on the upper basis of the cone and the difference between the cone height and the concrete which was in the cone was measured. The difference represented the magnitude of concrete slump, expressed in mm.
2. **Density of fresh concrete, EN 12350-6** is determined in a metal, cylindrical vessel with capacity of 8 dm^3. The vessel was filled with fresh concrete in two layers, each layer compacted by a metal rod. The

mass of the vessel before and after filling was measured. The difference of masses divided by the capacity of the vessel represented density of freshly compacted concrete.

3. **Air content in fresh concrete, EN 12350-7** was determined using the pressure method. The testing apparatus consisted of a metal cylindrical vessel with capacity of 8 dm^3 (the same vessel as the one for testing density of fresh concrete) and of the special lid with the pump and manometer. The vessel was filled with fresh concrete as in the case of previous testing of the density and then closed with the special lid. A special valve was used to to fill the space between the concrete surface and the lower side of the lid with water.After complete filling the valve was close. The pump was used to make the prescribed pressure in the cylinder under the lid, which then acted on the fresh concrete. Due to the pressure, water penetrated into concrete in places of air pores, and the manometer provided direct reading of the volume of the pores in relation to the volume of fresh concrete, i.e. vessel.
4. **Density of hardened concrete (water saturated), EN 12390-7** was determined on cube-shaped samples with dimensions of 150×150×150 mm. The mass of water saturated sample was measured, as well as the actual dimensions of the sample. The ratio between the mass and the volume obtained based on the measured dimensions represents density of hardened, water saturated concrete.
5. **Flexural strength, EN 12390-5** was tested on the prisms of 400 mm in length, having a square cross-section of 100×100 mm in dimensions. A digital hydraulic press "UTEST UTC - 5600" was used for the testing. A special tool that allows loading the sample with a bending force at a third of the span was used for the testing. The flexural strength was calculated according to the formula:

$$f_{zs,i} = \frac{6 \cdot M}{b \cdot h^2} = \left[\frac{N}{mm^2}, MPa\right]$$

where is the maximum flexural moment in kNm; b, h are width and height of the samples cross-section and $f_{zs,i}$ is tensile flexural strength in MPa.

6. **Compressive strength, EN 12390-3** was determined on the samples cube-shaped with dimensions of 150×150×150 mm. For the testing, digital, hydraulic press "UTEST UTC - 5740" with measuring range of 0-3000 kN and accuracy class of 0.5 % was used. The press was powered by servo-controlled hydraulic controller "UTC - 4860" and appropriate computer software. The test was conducted at the concrete samples with age of 2, 7, 28 and 90 days. Compressive strength was calculated according to the formula:

$$f_{p,i} = \frac{F_p}{A} = \left[\frac{N}{mm^2}, MPa\right]$$

where Fp is the pressure force leading to the failure of the specimen in N; A is surface are of the cube with a 150 mm side and $f_{p,i}$ is compressive strength of concrete in MPa.

7. **Tensile splitting strength, EN 12390-6** was determined on the concrete cylinder- shaped samples of 150 mm in diameter and 300 mm in hight. A digital hydraulic press "UTEST UTC - 5600" with accuracy class of 0.5 %, as well as a special tool allowing to load the sample set in horizontal position along the cylinder generatrix, were used for the testing. During the tests, constant increase of the load of 0.04 MPa/s was provided until the sample failure occured. Testing was conducted at the age of concrete samples of 28 days. Tensile splitting strength was calculated according to the formula:

$$f_{zc,i} = \frac{2 \cdot F_{zc}}{\pi \cdot d \cdot l} \quad \left[\frac{N}{mm^2}, MPa\right]$$

where F_{zc} is force causing the failure of the sample in N; d is cylinder diameter in mm; l is length (height) of the cylinder in mm and $f_{zc,i}$ is tensile splitting strength in MPa.

8. **Secant modulus of elasticity, EN 12390-13** was evaluated on the concrete cylindrical samples of 150 mm in diameter and 300 mm in hight at the age of 28 days. For the testing, digital, hydraulic press "UTEST UTC - 5740" with measuring range of 0-3000 kN and three digital extensometers "CONTROLS 55-C0222/F" was used. Immediately prior to testing, compressive strength of concrete was determined on 3 concrete specimens of same dimensions and age. Stabilized secant modulus of elasticity was determined according to the loading and unloading program, as shown in the following scheme.

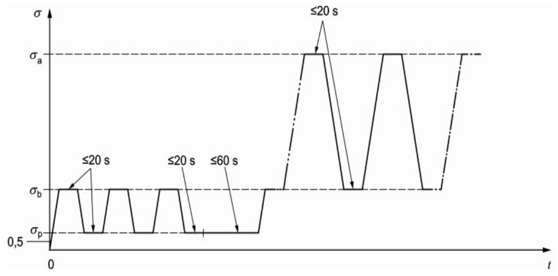

Legend:

_____ loading cycle,

__.._ loading cycle for the determination of initialsecant modulus of elasticity,

_ . __ loading cycle for the determination of stabilized secant modulus of elasticity,

σ applied stress in MPa,

σ_a upper stress fc/3,

σ_b lower stress $0.10\times fc \leq \sigma b \leq 0.15\times fc$,

σ_p preload stress 0.5 MPa $\leq \sigma p \leq \sigma b$,

t time in s.

9. **Dept of penetration of water under pressure, EN 12390-8** was tested on the concrete cylindrical samples of 150 mm in diameter and 300 mm in hight with age of more than 28 days. The samples were exposed to water under high pressure of 500 kPa for a period of 72 h. After that period, the samples were split and maximum depth of penetration of water in mm was measured. The water impermeability was tested on the device "UTEST UTC - 1090.92".
10. **Freeze-thaw resistance with de-icing salts – Scaling, CEN-TS_12390-9** was tested on the concrete samples in form of prisms with dimensions of 200 x 200 x 50 mm. At the age of samples of 25 days rubber was glued on all samples surfaces except for the surface of tested sample. At the age of concrete sample of 28 days, around 3 mm of 3 % NaCl solution of the temperature 20 °C was poured on the tested surface. The level of the of the NaCl solution was maintained by occasional adding of solution in the period of 72 h. Prior to the testing, all the sample surfaces except the tested one were insulated using the 20 mm thick styrofoam. The testing started at the age of samples of 31 days. Freeze-thaw resistance was assessed by measuring the mass of the scaled material after 56 cycles of alternating freezing/thawing. For each measuring and each sample, cumulative amount of scaled material (Sn) per unit of surface area after n cycles in kg/m^2 was calculated according to the equation:

$$S_n = \frac{m_{s,n}}{A} \cdot 10^3 \quad \left[\frac{kg}{m^2}\right]$$

where S_n is th mass of scaled material from the tested surface after n cycles in kg/m^2; $m_{s,n}$ is cumulative mass of dry scaled material after n cycle of thawing/freezing and A is the total tested surface area rounded to accuracy of 100 mm^2.
11. **Rebound number, EN 12504-2** was determined on the concrete cube-shaped samples, with sides of 100 mm, immediately prior to determining the compressive strength of specimens. Sclerometer "N-34 Proceq SA" Switzerland was used for the test. Prior to testing ach cubeic sample was loaded with a constant force of 50 kN using the hydraulic press. On two opposite sides of the sample, normally to the direction of placing of concrete, the rebound number Ri was measured at the angle of 0° at a total of ten locations. After reading the rebound number on the apparatus, the mean value of the rebound number was calculated, while making sure that no individual reading deviates more than 30 % from the mean value. The precise correlation between the rebound number and true compressive strength of concrete can be afterwards estimated.
12. **Ultrasonic pulse velocity, EN 12504-4)** was determined on the cube-shaped concrete samples with sides of 100 mm, immediately prior to determining the compressive strength of the specimens. Each concrete sample was tested on one point in the middle of the side. Ultrasonic apparatus "PUNDIT CNS Electronics LTD", with frequency of 54 kHz was used. The device display provided reading of time in μs required for the ultrasonic pulse to pass through the hardened concrete mass from the emitter to the receiver probes. Since the length of the impulse path is known, the velocity of ultrasonic pulse through concrete was determined based on the following formula:

$$V = \frac{L}{T} \quad \left[\frac{km}{s}\right]$$

where V is the ultrasonic pulse velocity in km/s; L is the path of ultrasonic pulse from the emitter to the receiver probe in mm; T is time required for the impulse to travel a path in μs.

The precise correlation between the ultrasonic pulse velocity and true compressive strength of concrete can be afterwards estimated

2.3.4. Preparing of the self-compacting concrete (SSC) mixtures and investigation of the parametars of self-compacting concrete (SSC) produced with the addition of waste sludge WSLP

For the purpose of standard parameters investigation, two different SCC mixtures were prepared: an etalon mixture as reference mix-design (marked with E-SCC; Table S3) and one mixture in which limestone filler was replaced with sludge WSLP (marked as WSLP-SCC; Table S3).

Table S3. The mix-design of the SCC produced with waste sludge WSLP as mineral admixture (November 2019).

E-SCC:	WSLP -SCC:
Cement 380 kg	WSLP -SCC: Cement 380 kg
Mineral admixture: limestone – 140 kg	Mineral admixture: waste material – 140 kg
River sand (0-4 mm) – 50% or 890 kg	River sand (0-4 mm) – 50% or 890 kg
Crushed aggregate (4/8 mm) – 15% or 267 kg	Crushed aggregate (4/8 mm) – 15% or 267 kg
Crushed aggregate (8/16 mm) – 35% or 623 kg	Crushed aggregate (8/16 mm) – 35% or 623 kg
Water 195.0 kg	Water 195.0 kg
Superplasticizer 4.6 kg	Superplasticizer 8.8 kg

Following parameters of self-compacting concrete (SCC) mixture produced with addition of the waste sludge WSLP from the wastewaters treatment plant of the powdery enamel, generated in November 2019 were determined with implementation of standard methods, as explained:

1. **Consistency – slump flow test, EN 12350-8** was determined using slump method typical for self compacting concretes (SCC) by means of the Abrams cone. Abrams cone of 30 cm in hight and 20 cm in diameter was placed on a flat surface, so that it was lying down on the lower base. The cone was completely filled with fresh concrete without any compacting. Than the cone was lifted upwards for 5 s and the concrete mixture was let to flow on the surface. When the concrete had completely settled, two diameters of concrete puddle were measured, and the mean value was value of slump.
2. **T_{500} test EN 12350-8** represents a parametar which was recorded as the time required for the self compacting concrete to slump to a diameter of 500 mm when running the slump flow test.
3. **L-box passing ratio (H_2/H_1), EN 12350-10)** was determering with the testing equipment that consisted of the L – box of rectangular cross section, with a horizontal and vertical part separated by a cover (outlet) in front of which was vertical reinforcement. Testing was performed in the following way: vertical section of the L – box was filled with concrete; then the cover was taken out so that the concrete coud flow from the vertical part into the horizontal section of the L – box (whereby the reinforcement resisted passage of concrete) and height of concrete at the beginning (H_1) and at the end (H_2) of the horizontal section of the L – box was measured. The ratio of heights H_2/H_1 represented the capacity of SCC for passing the obstacle.
4. **Segregation using sieves, EN 12350-11** was determined as a percentage of the amount of concrete which passed through the sieve with openings of 5 mm in relation to the total mass of concrete. For the testing, the sieve of 300 mm in diameter and 75 mm in height was used. On the sieve, 4.8 ± 0.2 kg of concrete was poured from the height of 50 ± 5 cm. After 2 min, the sieve was gently (without shaking) removed from the container on top of which it was placed, and the quantity of concrete that leaked out was measured. The percentage of concrete passing through the sieve was a measure of segregation.
5. **Density of fresh concrete, EN 12350-6**.
6. **Air content in fresh concrete, EN 12350-7**.

7. **Density of hardened concrete (water saturated), EN 12390-7**.
8. **Flexural strength, EN 12390-5**.
9. **Compressive strength, EN 12390-3**.
10. **Tensile splitting strength, EN 12390-6.**
11. **Secant modulus of elasticity, EN 12390-13**.
12. **Dept of penetration of water under pressure, EN 12390-8**.
13. **Freeze-thaw resistance with de-icing salts – Scaling, CEN-TS_12390-9**.
14. **Determination of rebound number, EN 12504-2**.
15. **Determination of ultrasonic pulse velocity, EN 12504-4.**
