# Supplementary material for: Sustainable Application of Waste Sludges from the Wastewater Treatment Plant Generated during the Production of Heating Devices in the Construction Industry
Source: Materials (Basel). 2024 Feb 27;17(5):1089. doi: 10.3390/ma17051089 (PMC10935120; doi:10.3390/ma17051089)
Supplement: Supplementary file 1 [file materials-17-01089-s001.zip › Supplementary Material S2.pdf]

Supplementary Materials S2

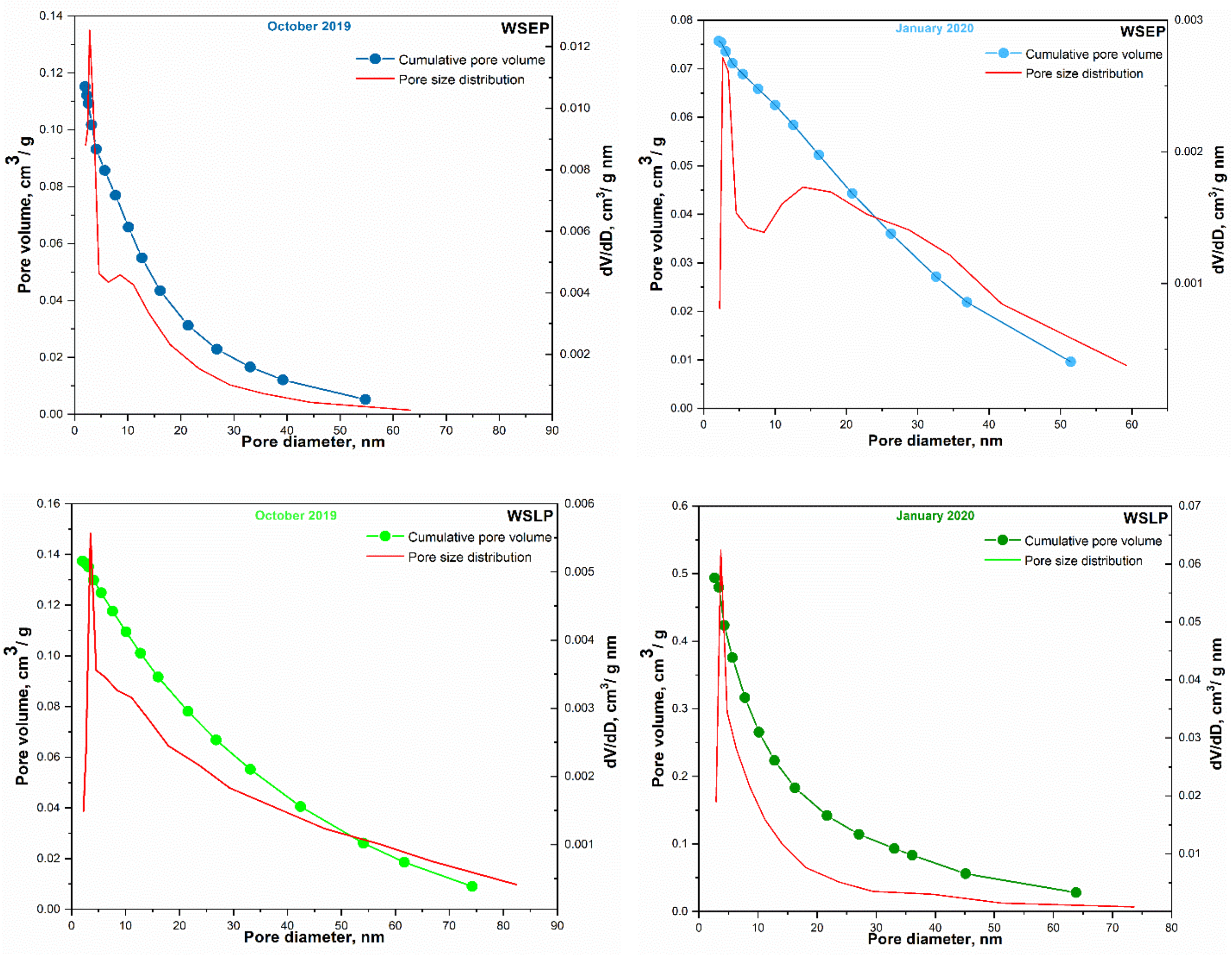

Figure S1. Total pore volume and pore size distribution by volume of the waste sludge WSEP generated in: (**a**) October 2019 and (**b**) January 2020; sludge WSLP generated in: (**c**) October 2019 and (**d**) January 2020.
