# Supplementary material for: Sustainable Application of Waste Sludges from the Wastewater Treatment Plant Generated during the Production of Heating Devices in the Construction Industry
Source: Materials (Basel). 2024 Feb 27;17(5):1089. doi: 10.3390/ma17051089 (PMC10935120; doi:10.3390/ma17051089)
Supplement: Supplementary file 1 [file materials-17-01089-s001.zip › Supplementary Material S3.pdf]

Supplementary Materials S3

4.1. The pozzolanic activity and the impact of cement replacement by waste sludges WSEP and WSLP on the cement paste properties

Table S4. Results of determination of pozzolanic activity and parameters of cement paste with addition of the waste sludge WSEP.

	**Class of pozzolanic materials**	**Activity index**	**Water requirement**	**Standard consistence**	**Initial setting time<br>Final setting time**	**Soundness**
**Standard**	**SRPS B.C1.018**	**EN 450-1<br>(25 % cement replacement)**	**EN 450-1 Annex B**	**EN 196-3<br>(25 % cement replacement)**	**EN 196-3<br>(25 % cement replacement)**	**EN 196-3<br>(30 % cement replacement)**
**October 2019**	Flexural strength – 2.05 MPa > 2.0 MPa (Requirements SRPS B.C1.018 p. 5.3 for class 5)	28 days – 77.61 % > 75 % (Requirements EN 450-1 p. 5.3.2)	102 % < 95 % (Requirements EN 450-1 p. 5.3.6)	33.5 % (100 % cement – 29.0 %) ((Not meet the condition for standard consistence)	300 min < 2× 100 % cement (100 % cement – 115 min) (Requirements EN 450-1 p. 5.3.5)	0.5 mm (100 % cement – 1.0 mm) < 10 mm (Requirements EN 450-1 p. 5.3.3)
	Compressive strength – 7.18 MPa > 5.0 MPa (Requirements SRPS B.C1.018 p. 5.3 for class 5)	90 days – 86.19 > 85 % (Requirements EN 450-1 p. 5.3.2)			330 min (100 % cement – 150 min) (Not meet the condition for final setting time)	
**Criteria**	Satisfies (Class 5 of pozzolanic materials)	Satisfies	Not satisfies	/	Not satisfies	Satisfies
	Satisfies (Class 5 of pozzolanic materials)	Satisfies			/	
**December 2019**	Flexural strength – 2.02 MPa > 2.0 MPa (Requirements SRPS B.C1.018 p. 5.3 for class 5)	28 days – 74.24 % > 75 % (Requirements EN 450-1 p. 5.3.2)	104 % < 95 % (Requirements EN 450-1 p. 5.3.6)	32.5 % (100 % cement – 29.0 %) ((Not meet the condition for standard consistence)	290 min < 2× 100 % cement (100 % cement – 115 min) (Requirements EN 450-1 p. 5.3.5)	0.5 mm (100 % cement – 1.0 mm) < 10 mm (Requirements EN 450-1 p. 5.3.3)
	Compressive strength – 5.55 MPa > 5.0 MPa (Requirements SRPS B.C1.018 p. 5.3 for class 5)	90 days – 85.26 > 85 % (Requirements EN 450-1 p. 5.3.2)			325 min (100 % cement – 150 min) (Not meet the condition for final setting time)	
**Criteria**	Satisfies (Class 5 of pozzolanic materials)	Not satisfies	Not satisfies	/	Not satisfies	Satisfies
	Satisfies (Class 5 of pozzolanic materials)	Satisfies			/	
**January 2020**	Flexural strength – 2.11 MPa > 2.0 MPa (Requirements SRPS B.C1.018 p. 5.3 for class 5)	28 days – 75.12 % > 75 % (Requirements EN 450-1 p. 5.3.2)	105 % < 95 % (Requirements EN 450-1 p. 5.3.6)	32.0 % (100 % cement – 29.0 %) ((Not meet the condition for standard consistence)	280 min < 2× 100 % cement (100 % cement – 115 min) (Requirements EN 450-1 p. 5.3.5)	0.5 mm (100 % cement – 1.0 mm) < 10 mm (Requirements EN 450-1 p. 5.3.3)
	Compressive strength – 5.67 MPa > 5.0 MPa (Requirements SRPS B.C1.018 p. 5.3 for class 5)	90 days – 85.76 > 85 % (Requirements EN 450-1 p. 5.3.2)			320 min (100 % cement – 150 min) (Not meet the condition for final setting time)	
**Criteria**	Satisfies (Class 5 of pozzolanic materials)	Satisfies	Not satisfies	/	Not satisfies	Satisfies
	Satisfies (Class 5 of pozzolanic materials)	Satisfies			/	

Table S5. Results of pozzolanic activity and parameters of cement paste with addition of the waste sludge WSLP.

	**Class of pozzolanic materials**	**Activity index**	**Water requirement**	**Standard consistence**	**Initial setting time**<br>**Final setting time**	**Soundness**
**Standard**	**SRPS B.C1.018**	**EN 450-1 (25 % cement replacement)**	**EN 450-1 Annex B**	**EN 196-3 (25 % cement replacement)**	**EN 196-3 (25 % cement replacement)**	**EN 196-3 (30 % cement replacement)**
**October 2019**	Flexural strength – 0.35 MPa > 2.0 MPa (Requirements SRPS B.C1.018 p. 5.3 for class 5)	28 days – 61.77 % > 75 % (Requirements EN 450-1 p. 5.3.2)	130 % < 95 % (Requirements EN 450-1 p. 5.3.6)	40.0 % (100 % cement – 29.0 %) ((Not meet the condition for standard consistence)	420 min < 2× 100 % cement (100 % cement – 115 min) (Requirements EN 450-1 p. 5.3.5)	3.0 mm (100 % cement – 1.0 mm) < 10 mm (Requirements EN 450-1 p. 5.3.3)
	Compressive strength – 0.83 MPa > 5.0 MPa (Requirements SRPS B.C1.018 p. 5.3 for class 5)	90 days – 70.15 > 85 % (Requirements EN 450-1 p. 5.3.2)			460 min (100 % cement – 150 min) (Not meet the condition for final setting time)	
**Criteria**	Not satisfies (without puzzolanic activity)	Not satisfies	Not satisfies	/	Not satisfies	Satisfies
	Not satisfies (without puzzolanic activity	Not satisfies			/	
**December 2019**	Flexural strength – 0.25 MPa > 2.0 MPa (Requirements SRPS B.C1.018 p. 5.3 for class 5)	28 days – 52.14 % > 75 % (Requirements EN 450-1 p. 5.3.2)	125 % < 95 % (Requirements EN 450-1 p. 5.3.6)	38.0 % (100 % cement – 29.0 %) ((Not meet the condition for standard consistence)	390 min < 2× 100 % cement (100 % cement – 115 min) (Requirements EN 450-1 p. 5.3.5)	2.5 mm (100 % cement – 1.0 mm) < 10 mm (Requirements EN 450-1 p. 5.3.3)
	Compressive strength – 0.58 MPa > 5.0 MPa (Requirements SRPS B.C1.018 p. 5.3 for class 5)	90 days – 60.84 > 85 % (Requirements EN 450-1 p. 5.3.2)			425 min (100 % cement – 150 min) (Not meet the condition for final setting time)	
**Criteria**	Not satisfies (without puzzolanic activity)	Not satisfies	Not satisfies	/	Not satisfies	Satisfies
	Not satisfies (without puzzolanic activity)	Not satisfies			/	
**January 2020**	Flexural strength – 0.12 MPa > 2.0 MPa (Requirements SRPS B.C1.018 p. 5.3 for class 5)	28 days – 50.26 % > 75 % (Requirements EN 450-1 p. 5.3.2)	128 % < 95 % (Requirements EN 450-1 p. 5.3.6)	40.0 % (100 % cement – 29.0 %) ((Not meet the condition for standard consistence)	420 min < 2× 100 % cement (100 % cement – 115 min) (Requirements EN 450-1 p. 5.3.5)	3.0 mm (100 % cement – 1.0 mm) < 10 mm (Requirements EN 450-1 p. 5.3.3)
	Compressive strength – 0.36 MPa > 5.0 MPa (Requirements SRPS B.C1.018 p. 5.3 for class 5)	90 days – 60.15 > 85 % (Requirements EN 450-1 p. 5.3.2)			460 min (100 % cement – 150 min) (Not meet the condition for final setting time)	
**Criteria**	Not satisfies (without puzzolanic activity)	Not satisfies	Not satisfies	/	Not satisfies	Satisfies
	Not satisfies (without puzzolanic activity)	Not satisfies			/	

4.2. Mortar parameters based on Portland cement, natural sand and added waste sludge WSEP

Table S6. Results of parameters of mortar mixtures based on Portland cement, natural sand and added waste sludge WSEP (October 2019).

**No.**	**Property**	Standard	Designation	Results
**1.**	**Consistency – by flow table**	EN 1015-3	E	135 mm
			WSEP-7.5	132 mm

			WSEP-15	128 mm
			WSEP-22.5	125 mm
			WSEP-30	118 mm
**2.**	**Bulk density of fresh mortar**	EN 1015-6	E	2299 kg/m^3
			WSEP-7.5	2294 kg/m^3
			WSEP-15	2289 kg/m^3
			WSEP-22.5	2363 kg/m^3
			WSEP-30	2259 kg/m^3
**3.**	**Dry bulk density of hardened mortar**	EN 1015-10	E	2294 kg/m^3
			WSEP-7.5	2290 kg/m^3
			WSEP-15	2284 kg/m^3
			WSEP-22.5	2360 kg/m^3
			WSEP-30	2256 kg/m^3
**4.**	**Flexural strength**	EN 196-1 (EN 1015-11)	E	6.0 MPa (2 days), 8.0 MPa (7 days), 8.1 MPa (28 days), 8.3 MPa (90 days)
			WSEP-7.5	5.8 MPa (2 days), 6.8 MPa (7 days), 8.3 MPa (28 days), 8.4 MPa (90 days)
			WSEP-15	5.2 MPa (2 days), 6.7 MPa (7 days), 7.5 MPa (28 days), 8.1 MPa (90 days)
			WSEP-22.5	5.1 MPa (2 days), 6.2 MPa (7 days), 7.1 MPa (28 days), 7.9 MPa (90 days)
			WSEP-30	4.3 MPa (2 days), 6.0 MPa (7 days), 6.6 MPa (28 days), 7.3 MPa (90 days)
**5.**	**Compressive strength**	EN 196-1 (EN 1015-11)	E	35.0 MPa (2 days), 52.7 MPa (7 days), 56.3 MPa (28 days), 63.0 MPa (90 days)
			WSEP-7.5	30.7 MPa (2 days), 40.4 MPa (7 days), 54.5 MPa (28 days), 62.2 MPa (90 days)
			WSEP-15	25.9 MPa (2 days), 39.2 MPa (7 days), 51.4 MPa (28 days), 57.9 MPa (90 days)
			WSEP-22.5	22.2 MPa (2 days), 34.8 MPa (7 days), 46.7 MPa (28 days), 56.3 MPa (90 days)
			WSEP-30	19.9 MPa (2 days), 33.6 MPa (7 days), 41.9 MPa (28 days), 50.1 MPa (90 days)
**6.**	**Water absorption at atmospheric pressure**	EN 13755	E	7.54%
			WSEP-7.5	7.87%
			WSEP-15	8.33%
			WSEP-22.5	8.92%
			WSEP-30	9.43%
**7.**	**Water absorption (Water absorption coefficient due to capillary action of hardened mortar)**	EN 1015-18		**Results given in the Table S7.**
**8.**	**Shrinkage**	SRPS B.C8.029:1979 (ASTM C 596)		**Results given in the Table S8.**
**9.**	**Adhesion a concrete substrate (Adhesive strength of hardened rendering and plastering mortars on substrates)**	EN 1015-12	E	2.0 N/mm^2
			WSEP-7.5	1.9 N/mm^2
			WSEP-15	1.7 N/mm^2
			WSEP-22.5	1.6 N/mm^2
			WSEP-30	1.5 N/mm^2

Table S7. Water absorption coefficient due to capillary action (C).

**Designation of mortar**	**Water absorption coefficient due to capillary action (C)**	
	For mortars other than renovation mortars [$kg/(m^2*min^{-0.5})$]	For renovation mortar [kg/m^2]
**E**	0.24	5.50

Table S8. Shrinkage due to drying over a period of 90 days.

**Designation of mortar**	**E**	**WSEP-7.5**	**WSEP-15**	**WSEP-22.5**	**WSEP-30**
**Age [days]**	$\varepsilon_{sm,sr}$ [mm/m]				
**3**	0.00	0.00	0.00	0.00	0.00

**WSEP-7.5**	0.24	5.85
**WSEP-15**	0.25	6.21
**WSEP-22.5**	0.27	6.64
**WSEP-30**	0.28	6.98

**4**	0.25	0.23	0.20	0.16	0.13
**7**	0.38	0.37	0.34	0.30	0.28
**14**	0.59	0.58	0.56	0.54	0.53
**21**	0.69	0.69	0.70	0.71	0.72
**28**	0.91	0.88	0.85	0.83	0.81
**56**	0.92	0.90	0.87	0.86	0.84
**90**	0.93	0.91	0.89	0.88	0.86

Table S9. Flexural and compressive strength of mortar mixtures based on Portland cement, natural sand and added waste sludge WSEP (November 2019).

4.	Flexural strength	EN 196-1 (EN 1015-11)	E	6.0 MPa (2 days), 8.0 MPa (7 days), 8.1 MPa (28 days), 8.3 MPa (90 days)
			WSEP-7.5	5.6 MPa (2 days), 6.7 MPa (7 days), 8.0 MPa (28 days), 8.3 MPa (90 days)
			WSEP-15	5.1 MPa (2 days), 6.5 MPa (7 days), 7.4 MPa (28 days), 8.1 MPa (90 days)
			WSEP-22.5	4.9 MPa (2 days), 6.1 MPa (7 days), 6.9 MPa (28 days), 7.8 MPa (90 days)
			WSEP-30	4.0 MPa (2 days), 5.8 MPa (7 days), 6.5 MPa (28 days), 7.3 MPa (90 days)
5.	Compressive strength	EN 196-1 (EN 1015-11)	E	35.0 MPa (2 days), 52.7 MPa (7 days), 56.3 MPa (28 days), 63.0 MPa (90 days)
			WSEP-7.5	30.0 MPa (2 days), 38.9 MPa (7 days), 53.1 MPa (28 days), 60.8 MPa (90 days)
			WSEP-15	25.9 MPa (2 days), 37.0 MPa (7 days), 50.2 MPa (28 days), 56.7 MPa (90 days)
			WSEP-22.5	22.2 MPa (2 days), 33.3 MPa (7 days), 44.9 MPa (28 days), 54.6 MPa (90 days)
			WSEP-30	19.9 MPa (2 days), 31.9 MPa (7 days), 40.2 MPa (28 days), 48.6 MPa (90 days)

Table S10. Water absorption coefficient due to capillary action (C).

**Designation of mortar**	**Water absorption coefficient due to capillary action (C)**	
	For mortars other than renovation mortars [kg/(m^2*min$^{-0.5}$)]	For renovation mortar [kg/m^2]
**E**	0.24	5.50
**WSEP-7.5**	0.25	5.82
**WSEP-15**	0.26	6.15
**WSEP-22.5**	0.26	6.58

Table S11. Shrinkage due to drying over a period of 90 days.

**Designation of mortar**	**E**	**WSEP-7.5**	**WSEP-15**	**WSEP-22.5**	**WSEP-30**
**Age [days]**	$\varepsilon_{sm,sr}$ [mm/m]				
**3**	0.00	0.00	0.00	0.00	0.00
**4**	0.25	0.23	0.20	0.17	0.15
**7**	0.38	0.37	0.35	0.34	0.33
**14**	0.59	0.59	0.58	0.58	0.57

**WSEP-30**	0.28	6.87

**21**	0.69	0.70	0.72	0.73	0.75
**28**	0.91	0.90	0.88	0.86	0.84
**56**	0.92	0.92	0.90	0.88	0.86
**90**	0.93	0.93	0.92	0.90	0.88

Table S12. Results of parameters of mortar mixtures based on Portland cement, natural sand and added waste sludge WSEP (December 2019).

No.	Property	Standard	Designation	Results
**1.**	**Consistency – by flow table**	EN 1015-3	E	135 mm
			WSEP-7.5	133 mm
			WSEP-15	130 mm
			WSEP-22.5	124 mm
			WSEP-30	119 mm
**2.**	**Bulk density of fresh mortar**	EN 1015-6	E	2299 kg/m^3
			WSEP-7.5	2294 kg/m^3
			WSEP-15	2286 kg/m^3
			WSEP-22.5	2372 kg/m^3
			WSEP-30	2262 kg/m^3
**3.**	**Dry bulk density of hardened mortar**	EN 1015-10	E	2294 kg/m^3
			WSEP-7.5	2288 kg/m^3
			WSEP-15	2281 kg/m^3
			WSEP-22.5	2368 kg/m^3
			WSEP-30	2258 kg/m^3
**4.**	**Flexural strength**	EN 196-1 (EN 1015-11)	E	6.0 MPa (2 days), 8.0 MPa (7 days), 8.1 MPa (28 days), 8.3 MPa (90 days)
			WSEP-7.5	5.4 MPa (2 days), 6.5 MPa (7 days), 7.8 MPa (28 days), 8.1 MPa (90 days)
			WSEP-15	5.0 MPa (2 days), 6.2 MPa (7 days), 7.3 MPa (28 days), 7.9 MPa (90 days)
			WSEP-22.5	4.7 MPa (2 days), 6.0 MPa (7 days), 6.7 MPa (28 days), 7.5 MPa (90 days)
			WSEP-30	3.9 MPa (2 days), 5.5 MPa (7 days), 6.2 MPa (28 days), 7.0 MPa (90 days)
**5.**	**Compressive strength**	EN 196-1 (EN 1015-11)	E	35.0 MPa (2 days), 52.7 MPa (7 days), 56.3 MPa (28 days), 63.0 MPa (90 days)
			WSEP-7.5	30.0 MPa (2 days), 37.8 MPa (7 days), 51.7 MPa (28 days), 59.9 MPa (90 days)
			WSEP-15	25.1 MPa (2 days), 35.8 MPa (7 days), 49.1 MPa (28 days), 56.0 MPa (90 days)
			WSEP-22.5	21.5 MPa (2 days), 32.1 MPa (7 days), 43.5 MPa (28 days), 53.7 MPa (90 days)
			WSEP-30	19.4 MPa (2 days), 30.2 MPa (7 days), 39.7 MPa (28 days), 48.3 MPa (90 days)
**6.**	**Water absorptionat atmospheric pressure)**	EN 13755	E	7.54%
			WSEP-7.5	7.93%
			WSEP-15	8.38%
			WSEP-22.5	8.94%
			WSEP-30	9.41%
**7.**	**Water absorption (Water absorption coefficient due to capillary action of hardened mortar)**	EN 1015-18		**Results given in the Table S13.**
**8.**	**Shrinkage**	SRPS B.C8.029:1979 (ASTM C 596)		**Results given in the Table S14.**

**9.**	**Adhesion a concrete substrate (Adhesive strength of hardened rendering and plastering mortars on substrates)**	EN 1015-12	E	2.0 N/mm^2
			WSEP-7.5	1.8 N/mm^2
			WSEP-15	1.6 N/mm^2
			WSEP-22.5	1.5 N/mm^2
			WSEP-30	1.5 N/mm^2

Table S13. Water absorption coefficient due to capillary action (C).

**Designation of mortar**	**Water absorption coefficient due to capillary action (C)**	
	For mortars other than renovation mortars [kg/(m^2*min$^{-0.5}$)]	For renovation mortar [kg/m^2]
**E**	0.24	5.50
**WSEP-7.5**	0.25	5.86
**WSEP-15**	0.27	6.21
**WSEP-22.5**	0.28	6.64
**WSEP-30**	0.29	6.92

Table S14. Shrinkage due to drying over a period of 90 days.

**Designation of mortar**	**E**	**WSEP-7.5**	**WSEP-15**	**WSEP-22.5**	**WSEP-30**
**Age [days]**	$\varepsilon_{sm,sr}$ [mm/m]				
**3**	0.00	0.00	0.00	0.00	0.00
**4**	0.25	0.23	0.19	0.17	0.14
**7**	0.38	0.37	0.35	0.33	0.32
**14**	0.59	0.58	0.59	0.58	0.59
**21**	0.69	0.70	0.72	0.72	0.73
**28**	0.91	0.89	0.86	0.84	0.82
**56**	0.92	0.90	0.88	0.87	0.85
**90**	0.93	0.92	0.91	0.89	0.87

Table S15. Results of parameters of mortar mixtures based on Portland cement, natural sand and added waste sludge WSEP (January 2020).

**No.**	**Property**	Standard	Designation	Results
**1.**	**Consistency – by flow table**	EN 1015-3	E	135 mm
			WSEP-7.5	132 mm
			WSEP-15	128 mm
			WSEP-22.5	123 mm
			WSEP-30	117 mm
**2.**	**Bulk density of fresh mortar**	EN 1015-6	E	2299 kg/m^3
			WSEP-7.5	2291 kg/m^3
			WSEP-15	2282 kg/m^3
			WSEP-22.5	2370 kg/m^3
			WSEP-30	2257 kg/m^3
**3.**	**Dry bulk density of hardened mortar**	EN 1015-10	E	2294 kg/m^3
			WSEP-7.5	2287 kg/m^3
			WSEP-15	2278 kg/m^3
			WSEP-22.5	2367 kg/m^3

			WSEP-30	2254 kg/m^3
4.	**Flexural strength**	EN 196-1 (EN 1015-11)	E	6.0 MPa (2 days), 8.0 MPa (7 days), 8.1 MPa (28 days), 8.3 MPa (90 days)
			WSEP-7.5	5.5 MPa (2 days), 6.7 MPa (7 days), 7.9 MPa (28 days), 8.2 MPa (90 days)
			WSEP-15	5.0 MPa (2 days), 6.3 MPa (7 days), 7.5 MPa (28 days), 8.0 MPa (90 days)
			WSEP-22.5	4.8 MPa (2 days), 6.1 MPa (7 days), 6.8 MPa (28 days), 7.7 MPa (90 days)
			WSEP-30	4.0 MPa (2 days), 5.7 MPa (7 days), 6.4 MPa (28 days), 7.2 MPa (90 days)
5.	**Compressive strength**	EN 196-1(EN 1015-11)	E	35.0 MPa (2 days), 52.7 MPa (7 days), 56.3 MPa (28 days), 63.0 MPa (90 days)
			WSEP-7.5	31.3 MPa (2 days), 38.4 MPa (7 days), 52.7 MPa (28 days), 59.9 MPa (90 days)
			WSEP-15	25.9 MPa (2 days), 36.7 MPa (7 days), 49.5 MPa (28 days), 57.1 MPa (90 days)
			WSEP-22.5	22.4 MPa (2 days), 33.4 MPa (7 days), 43.3 MPa (28 days), 53.8 MPa (90 days)
			WSEP-30	20.2 MPa (2 days), 31.0 MPa (7 days), 40.4 MPa (28 days), 49.0 MPa (90 days)
6.	**Water absorption at atmospheric pressure**	EN 13755	E	7.54%
			WSEP-7.5	8.02%
			WSEP-15	8.47%
			WSEP-22.5	9.11%
			WSEP-30	9.64%
7.	**Water absorption (Water absorption coefficient due to capillary action of hardened mortar)**	EN 1015-18		**Results given in the Table S16.**
8.	**Shrinkage**	SRPS B.C8.029:1979 (ASTM C 596)		**Results given in the Table S17.**
9.	**Adhesion a concrete substrate (Adhesive strength of hardened rendering and plastering mortars on substrates)**	EN 1015-12	E	2.0 N/mm^2
			WSEP-7.5	1.8 N/mm^2
			WSEP-15	1.7 N/mm^2
			WSEP-22.5	1.6 N/mm^2
			WSEP-30	1.5 N/mm^2

Table S16. Water absorption coefficient due to capillary action (C).

Designation of mortar	Water absorption coefficient due to capillary action (C)	
	For mortars other than renovation mortars [kg/(m^2*min$^{-0.5}$)]	For renovation mortar [kg/m^2]
**E**	0.24	5.50
**WSEP-7.5**	0.26	5.94
**WSEP-15**	0.28	6.30
**WSEP-22.5**	0.29	6.72
**WSEP-30**	0.31	7.04

Table S17. Shrinkage due to drying over a period of 90 days.

Designation of mortar	E	WSEP-7.5	WSEP-15	WSEP-22.5	WSEP-30
**Age [days]**	$\varepsilon_{sm,sr}$ [mm/m]				
**3**	0.00	0.00	0.00	0.00	0.00
**4**	0.25	0.22	0.18	0.15	0.13
**7**	0.38	0.36	0.34	0.31	0.30
**14**	0.59	0.59	0.57	0.56	0.56
**21**	0.69	0.70	0.71	0.72	0.72
**28**	0.91	0.88	0.86	0.83	0.80
**56**	0.92	0.90	0.88	0.86	0.83
**90**	0.93	0.92	0.90	0.88	0.85
